# Regulation of DNA damage response by trimeric G-proteins

**DOI:** 10.1016/j.isci.2023.105973

**Published:** 2023-01-13

**Authors:** Amer Ali Abd El-Hafeez, Nina Sun, Anirban Chakraborty, Jason Ear, Suchismita Roy, Pranavi Chamarthi, Navin Rajapakse, Soumita Das, Kathryn E. Luker, Tapas K. Hazra, Gary D. Luker, Pradipta Ghosh

**Affiliations:** 1 Department of Cellular and Molecular Medicine, University of California San Diego, La Jolla, CA 92093, USA; 2 Department of Internal Medicine, University of Texas Medical Branch, Galveston, TX 77555, USA; 3 Biological Sciences Department, California State Polytechnic University, Pomona, CA 91768, USA; 4 Department of Pathology, University of California San Diego, La Jolla, CA 92093, USA; 5 Center for Molecular Imaging, Department of Radiology, University of Michigan, 109 Zina Pitcher Place, Ann Arbor, MI 48109-2200, USA; 6 Department of Biomedical Engineering, University of Michigan, 2200 Bonisteel, Blvd., Ann Arbor, MI 48109-2099, USA; 7 Department of Microbiology and Immunology, University of Michigan, 109 Zina Pitcher Place, Ann Arbor, MI 48109-2200, USA; 8 Department of Medicine, University of California San Diego, La Jolla, CA 92093, USA; 9 Moores Comprehensive Cancer Center, University of California San Diego, La Jolla, CA 92093, USA; 10 Veterans Affairs Medical Center, La Jolla, CA, USA; 11 Pharmacology and Experimental Oncology Unit, Cancer Biology Department, National Cancer Institute, Cairo University, Cairo, Egypt

**Keywords:** Biological sciences, Cell biology, Molecular biology

## Abstract

Upon sensing DNA double-strand breaks (DSBs), eukaryotic cells either die or repair DSBs via one of the two competing pathways, i.e., non-homologous end-joining (NHEJ) or homologous recombination (HR). We show that cell fate after DSBs hinges on GIV/Girdin, a guanine nucleotide-exchange modulator of heterotrimeric Giα•βγ protein. GIV suppresses HR by binding and sequestering BRCA1, a key coordinator of multiple steps within the HR pathway, away from DSBs; it does so using a C-terminal motif that binds BRCA1’s BRCT-modules via both phospho-dependent and -independent mechanisms. Using another non-overlapping C-terminal motif GIV binds and activates Gi and enhances the “free” Gβγ→PI-3-kinase→Akt pathway, which promotes survival and is known to suppress HR, favor NHEJ. Absence of GIV, or loss of either of its C-terminal motifs enhanced cell death upon genotoxic stress. Because GIV selectively binds other BRCT-containing proteins suggests that G-proteins may fine-tune sensing, repair, and survival after diverse types of DNA damage.

## Highlights

• Non-receptor G protein modulator, GIV/Girdin binds BRCA1
• Binding occurs in both canonical and non-canonical modes
• GIV sequesters BRCA1 away from dsDNA breaks, suppresses HR
• Activation of Gi by GIV enhances Akt, suppresses HR, favors NHEJ

## Graphical abstract

**Figure:**
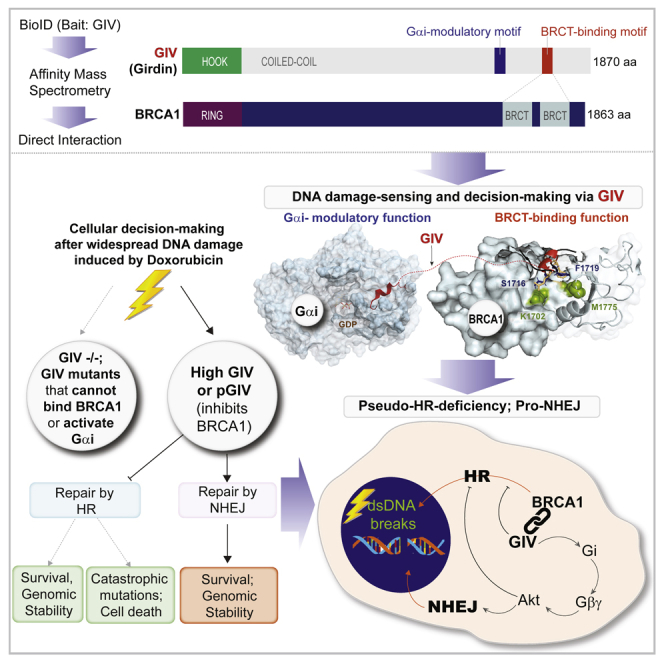

## Introduction

Genomic integrity is under constant attack from extrinsic and intrinsic factors that induce DNA damage.^1^ Damaged DNA must be repaired to maintain genomic integrity via processes that evolved by the cell type, collectively termed the DNA damage response (DDR).^2^ DDRs are orchestrated by an incredibly complex network of proteins that sense and assess the type and extent of damage, decide between cell fates (death vs. repair), choose a repair pathway, and then initiate and complete the repair process.^3^ For example, the DDR involves the activation of ATM kinase, a member of the phosphoinositide 3-kinase (PI3K)-related protein kinase family^4^ which is rapidly recruited by the MRE11-RAD50-NBS1 (MRN) complex to chromatin.^5^ Phosphorylation of a large number of substrates follows, which in turn activates cell cycle checkpoints and triggers the recruitment of repair factors to the DSBs. Positive feedback loops are orchestrated to amplify the signals, e.g., ATM phosphorylates the histone variant H2AX (resulting in the formation of the phosphorylated form called γH2AX),^6^ which recruits additional ATM molecules and further accumulation of γH2AX.^7^^,^^8^^,^^9^

Among the types of DNA damage, DNA double-strand breaks (DSBs) are the most cytotoxic lesions that threaten genomic integrity.^10^ Failure to repair DSBs results in genomic instability and cell death. DNA repair can be achieved by different means that are commonly grouped into two broad, competing categories:^11^ homologous recombination (HR) and non-homologous end-joining (NHEJ).^12^ HR, which requires a homologous template to direct DNA repair, is generally believed to be a high-fidelity pathway.^13^ By contrast, NHEJ directly seals broken ends; while some believe that repair by NHEJ is imprecise, we have shown that precision can indeed be achieved.^14^ In fact, NHEJ offers an ideal balance of flexibility and accuracy when the damage to DNA is widespread with DSBs featuring diverse end structures.^15^ Consequently, it is believed to represent the simplest and fastest mechanism to heal DSBs,^16^ thus it is the most predominant DSB repair pathway within the majority of mammalian cells. Key molecular players in both pathways have been identified: 53BP1, first identified as a DNA damage checkpoint protein, and Breast cancer type 1 susceptibility protein (BRCA1), a well-known breast cancer tumor suppressor,^17^ are at the center of molecular networks that coordinate NHEJ and HR, respectively.

How the choice of DSB repair pathway is determined at a molecular level has been the subject of intense study for a decade.^18^^,^^19^ Here we reveal a previously unforeseen determinant of the choice of DNA damage, Gα-Interacting Vesicle-associated protein (GIV; also known as, Girdin), which is a non-receptor activator of heterotrimeric (henceforth, trimeric) G-protein, Gi^20^^,^^21^ Trimeric G-proteins are a major signaling hub in eukaryotes that gate signaling downstream of 7-transmembrane (7TM)-receptors called GPCRs, and the GPCR/G-protein pathway is of paramount importance in modern medicine, serving as a target of about 34% of marketed drugs.^22^ Although peripheral players in the GPCR/G-protein pathway have been found to have indirect impact on DDR [reviewed in^23^], the role of G-proteins in DDR has never been established. Unlike GPCRs that primarily sense the exterior of the cell, GIV-GEM the prototypical member of a family of cytosolic guanine-nucleotide exchange modulators (GEMs), senses and coordinates cellular response to intracellular events (e.g., autophagy, ER-stress, unfolded protein response, inside-out signaling during mechanosensing, and so forth) by activating endomembrane localized GTPases.^24^^,^^25^ By virtue of its ability to coordinate multiple cellular processes, many of which impart aggressive traits to tumor cells, GIV has emerged as a *bona-fide* oncogene that supports cancer cell stemness, emergence of chemoresistance and invasion, favors aggressive tumor phenotypes in diverse types of cancers, and drives poor survival utcomes [reviewed in^26^]. We provide mechanistic insights into how GIV-GEM binds and inhibits BRCA1, a regulator of multiple steps within the HR pathway, and concomitantly enhances Akt signaling; the latter is a key component within the NHEJ pathway. In doing so, this work not only reveals another pro-tumorigenic role of GIV but also begins to unravel how endomembrane G-protein signaling shapes the cellular response to DNA damage.

## Results

### Proteomic studies suggest a putative role for GIV during DNA damage response

**Figure 1:**
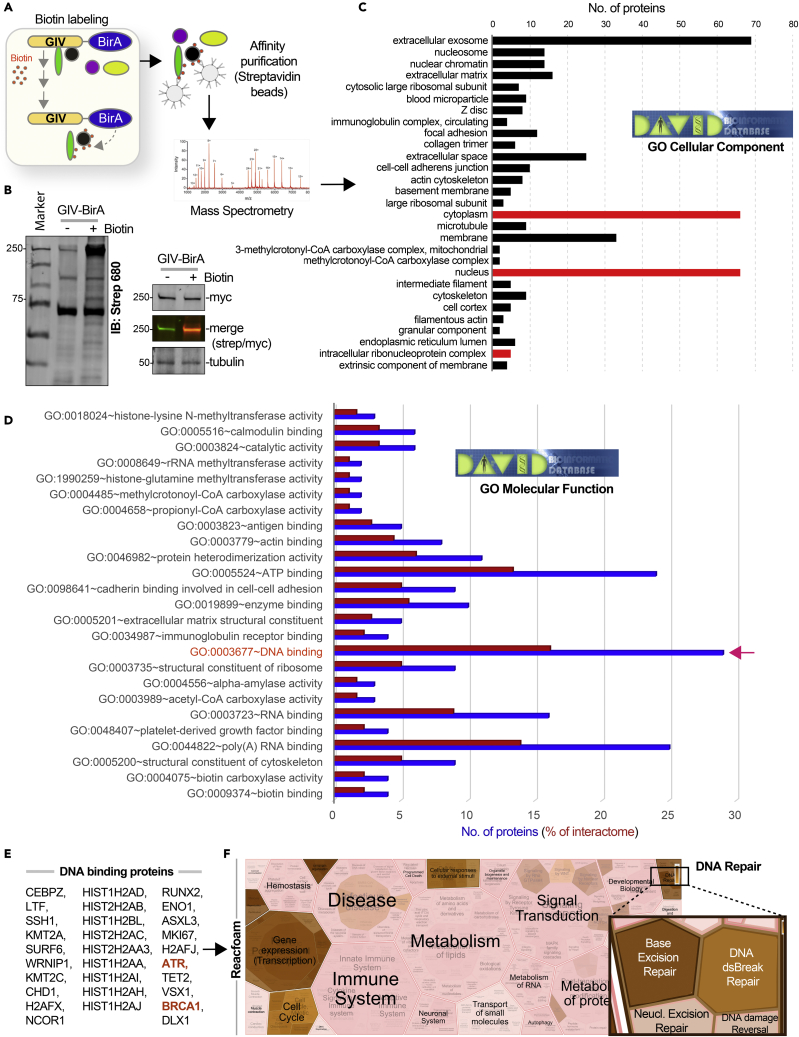
Proteomic studies suggest an intranuclear role of GIV/Girdin in DNA damage repair response (A) Schematic outlining key steps in BioID studies to identify the GIV interactome. (B) Immunoblots confirm biotinylation in HEK whole cell lysates (left) and expression of the BirA-tagged full-length GIV construct as a protein of expected size (right). (C and D) Bar plots show GO analyses [cellular component (C) and molecular function (D)] for bioID-identified GIV interactome. Red bars in C indicate putative compartments where GIV binds BRCA1. Blue and red bars in D indicate a total number of interacting proteins and % representation, respectively. Red arrow in D indicates the molecular function category where BRCA1 was identified. (E and F) DNA-binding proteins (listed in E) that were identified in GIV’s interactome were analyzed by Reactome.org and visualized as hierarchical reacfoam (in F). Inset in the top right corner is magnified to highlight the overrepresentation of DNA repair pathways.

The interactome of a protein dictates its localization and cellular functions. To map the landscape of GIV’s interactome we carried out proximity-dependent biotin identification (BioID) coupled with mass spectrometry (MS) (Figure 1A). BirA-tagged GIV construct was validated by immunoblotting and found to be expressed in cells as full-length proteins of expected molecular size (∼250 kDa) (Figure 1B). Samples were subsequently processed for protein identification by Mass Spectrometry. Gene ontology (GO) cellular component analysis, as determined by DAVID GO, revealed that GIV-proximal interactors were in both cytoplasmic and nuclear compartments (Figure 1C; See Table S1); one interactor (i.e., BRCA1) was predicted to bind GIV across three different cellular compartments (Figure 1C; red bars). GO-molecular function analysis revealed that “DNA binding proteins” was the most enriched class of proteins in GIV’s interactome (Figure 1D and Table S2). BRCA1 was a notable interactor within that class, and the serine/threonine-specific protein kinase, Ataxia telangiectasia, and Rad3-related protein (ATR), that coordinates DNA damage sensing and repair was another (Figure 1E). A reactome pathway analysis of DNA-binding proteins in the GIV’s interactome showed that GIV’s interactome enrichment for proteins that participate in gene transcription, regulation of the cell cycle, and in DNA repair (Figures 1E and 1F). It is noteworthy that although GIV’s presence on nuclear speckles was described almost a decade ago,^27^ little is known about GIV’s role in sensing/signaling during intranuclear processes.

### GIV is required for DNA damage response

**Figure 2:**
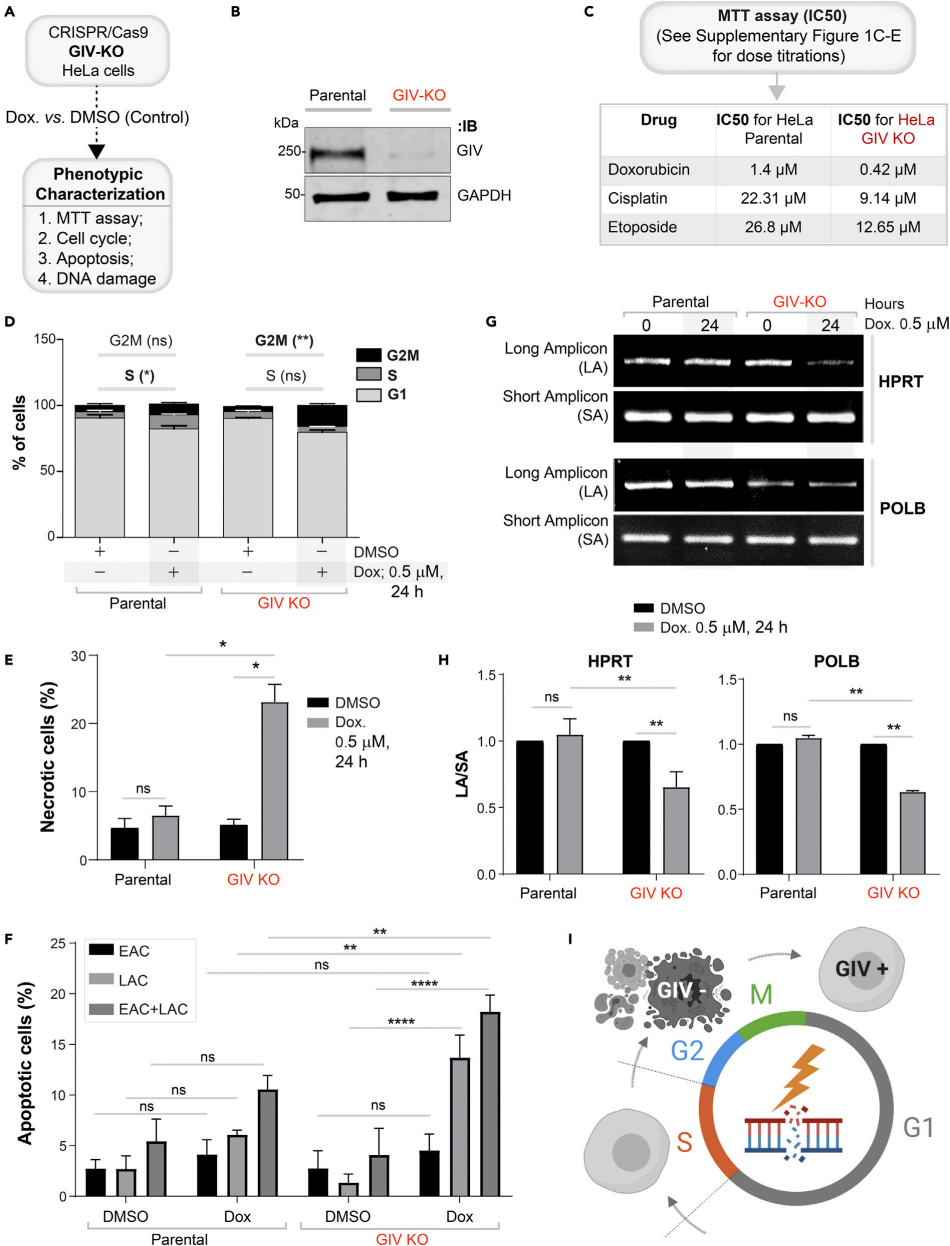
DNA damage repair response is impaired in cells without GIV (A) Schematic outlining the cell lines and phenotypic assays displayed in this figure. (B) Immunoblot of GIV-depleted (by CRISPR Cas9) and control (Parental) HeLa cell lysates showing the depletion of full-length endogenous GIV. See also Figure S1 for how pooled KO lines were generated. (C) Table of IC50 values for 3 different drugs tested on parental and GIV KO HeLa cells, as determined using MTT assays. See Figures S1C and S1E for the dose-dependent survival curves. (D) Stacked bar graphs showing the percentage of cells at various stages of the cell cycle (G1, S, and G2/M) after challenged with Dox or vehicle control (DMSO). Histograms are shown in Figure S1F. Data displayed as mean ± SEM and one-way ANOVA using Tukey’s multiple comparisons test was used to determine significance. (∗; p ≤ 0.05, ∗∗; p ≤ 0.01; ns = not significant). (E and F) Bar graphs display the % necrotic (E) or apoptotic (early, EAC; late, LAC; or combined) cells after challenged with either Dox or vehicle control (DMSO), as assessed by annexin V staining and flow cytometry. See Figure S1G for the dot plot diagrams. (G and H) Long amplicon qPCR (LA-QPCR) was used to evaluate genomic DNA SB levels in control vs. GIV KO cells. Representative gel showing PCR-amplified fragments of the *HPRT* (G, top panel) and *POLB* (G, bottom panel) genes. Amplification of each large fragment (upper panels) was normalized to that of a small fragment of the corresponding gene (bottom panels), and the data were expressed as normalized (with short PCR amplicon) relative band intensity with the DMSO-treated (0 h) sample in each case arbitrarily set as unity and displayed as a bar graph in H. Full-length gels can be seen in Figure S1H. Data displayed as mean ± SEM and one-way ANOVA to determine significance. (∗∗; p ≤ 0.01; ∗∗∗∗; p ≤ 0.0001; ns = not significant). (I) Summary of the phenotype of cells with (parental; GIV +) or without GIV (GIV KO; GIV -). See also Table S3.

We generated HeLa cells without GIV using CRISPR Cas9 and subsequently exposed them to Doxorubicin followed by several commonly used readouts of DDR (Figures 2A, S1A, and S1B). A mixture of −/− (henceforth, GIV KO) clones was pooled to recapitulate the clonal heterogeneity of parental HeLa cells (Figure S1B), and near-complete depletion of GIV (estimated ∼95% by band densitometry) was confirmed by immunoblotting (Figure 2B). We chose HeLa cells because DDR has been extensively studied in this cell line^28^^,^^29^ and because HeLa cells have defective p53.^30^ The latter is relevant because GIV/CCDC88A aberrations (gene amplification) co-occur with defects in the tumor suppressor TP53 (TCGA pancancer profile; cbioportal.org); ∼36% of tumors with aberrant CCDC88A expression was also associated with mis-sense and truncating driver mutations in TP53. We chose Doxorubicin (henceforth, Dox) for inducing DNA damage because it is a widely used anthracycline anticancer agent and its impact on DNA integrity in HeLa cells has been mapped for each cell cycle with demonstrated reproducibility.^31^ Compared to parental cells, fewer metabolically active GIV KO cells survived after a Dox challenge, as determined using an MTT assay (Figures 2C and S1C), indicating that in the absence of GIV, cells show markedly reduced survival from cytotoxic lesions induced by Dox. GIV KO cells showed increased susceptibility also to two other cytotoxic drugs, Cisplatin and Etoposide (Figures S1D and S1E). The lower IC50 values in the case of GIV KO cell lines for all 3 drugs (Figure 2C) imply that GIV is required for surviving cytotoxic lesions induced by the most commonly used cytotoxic drugs.

Because cell cycle is a key determinant of the choice of repair pathway, next, we asked if GIV may impact one or more of the three checkpoints (G1/S, S phase, and G2/M) where cell cycle may be arrested in response to DNA damage. We found that Dox-challenged parental cells, as expected for cells with defective p53, escaped the G1/S checkpoint,^32^ and instead, preferentially showed arrest in the S/G2 phase; however, GIV KO cells showed no such S phase arrest and instead arrested in the G2 phase (Figures 2D and S1F). Because chromosome duplication occurs during the "S phase" (the phase of DNA synthesis) and this phase surveys DNA for replication errors,^33^ failure of GIV KO cells to arrest in the S phase indicates that this “checkpoint” is impaired (i.e., bypassed). Because irreparable DNA injury leads to the accumulation of mutations, which in turn may induce either apoptosis or necrosis,^34^ next we analyzed cell death by flow cytometry using a combination of annexin V and propidium iodide (PI) staining. Compared to parental control cells, Dox challenge induced a significantly higher rate of cell death in GIV KO cells (Figures 2E, 2F, and S1G), via both necrosis (Figure 2E) and apoptosis (specifically, late apoptosis; Figure 2F).

To examine whether higher cell death was related to impaired repair activity and an accumulation of DNA strand breaks in GIV KO cells, genomic DNA was isolated from parental and GIV KO cells, with or without Dox challenge and the levels of strand breaks in the *HPRT* and *POLB* genes were compared using long amplicon qPCR (LA-qPCR) as described previously.^35^ Strand breaks were measured for both the genes using a Poisson distribution, and the results were expressed as the lesion/10 kb genome.^36^ A decreased level of the long amplicon PCR product (12.2 kb of the POLB or 10.4 kb region of the HPRT gene) would reflect a higher level of breaks; however, the amplification of a smaller fragment for each gene is expected to be similar for the samples, because of a lower probability of breaks within a shorter fragment. A higher level of DNA strand break was observed in the genomic DNA of GIV KO cells than in the DNA of parental controls (Figures 2G, 2H, and S1H), indicating a role of GIV in DNA repair.

Reduced cell survival (Figure 2C), cell-cycle arrest (Figure 2D), higher cell death (Figures 2E and 2F), and the accumulation of cytotoxic lesions (Figures 2G and 2H) in GIV KO cells were also associated with reduced growth in anchorage-dependent clonogenic growth assays (Figure S1I).

To test if the pro-survival functions of GIV in the setting of cytotoxic lesions are cell-type specific, we compared 2 other cell lines, the MDA-MB-231breast and DLD-1 colorectal cancer lines (Figure S2A). We generated GIV KO MDA-MB-231 cell lines using CRISPR Cas9 (see validation in Figures S2B and S2C) and used the previously validated GIV KO DLD-1 cells.^37^ We exposed these cells to Doxorubicin. Survival was significantly impaired in all the GIV KO cell lines (Figures S2D–S2F), implying that our findings in HeLa cells may be broadly relevant in diverse cancers.

Taken together, these findings demonstrate that GIV is required for DNA repair; in cells without GIV, cell survival is reduced, S phase checkpoint is lost, and DNA repair is impaired, leading to the accumulation of mutations in *POLB* and *HPRT* (see Figure 2I). These findings suggest that genotoxic insult in the absence of GIV may lead to the accumulation of catastrophic amounts of mutations that may ultimately trigger cell death.

### The C-terminus of GIV binds tandem BRCT modules of BRCA1

**Figure 3:**
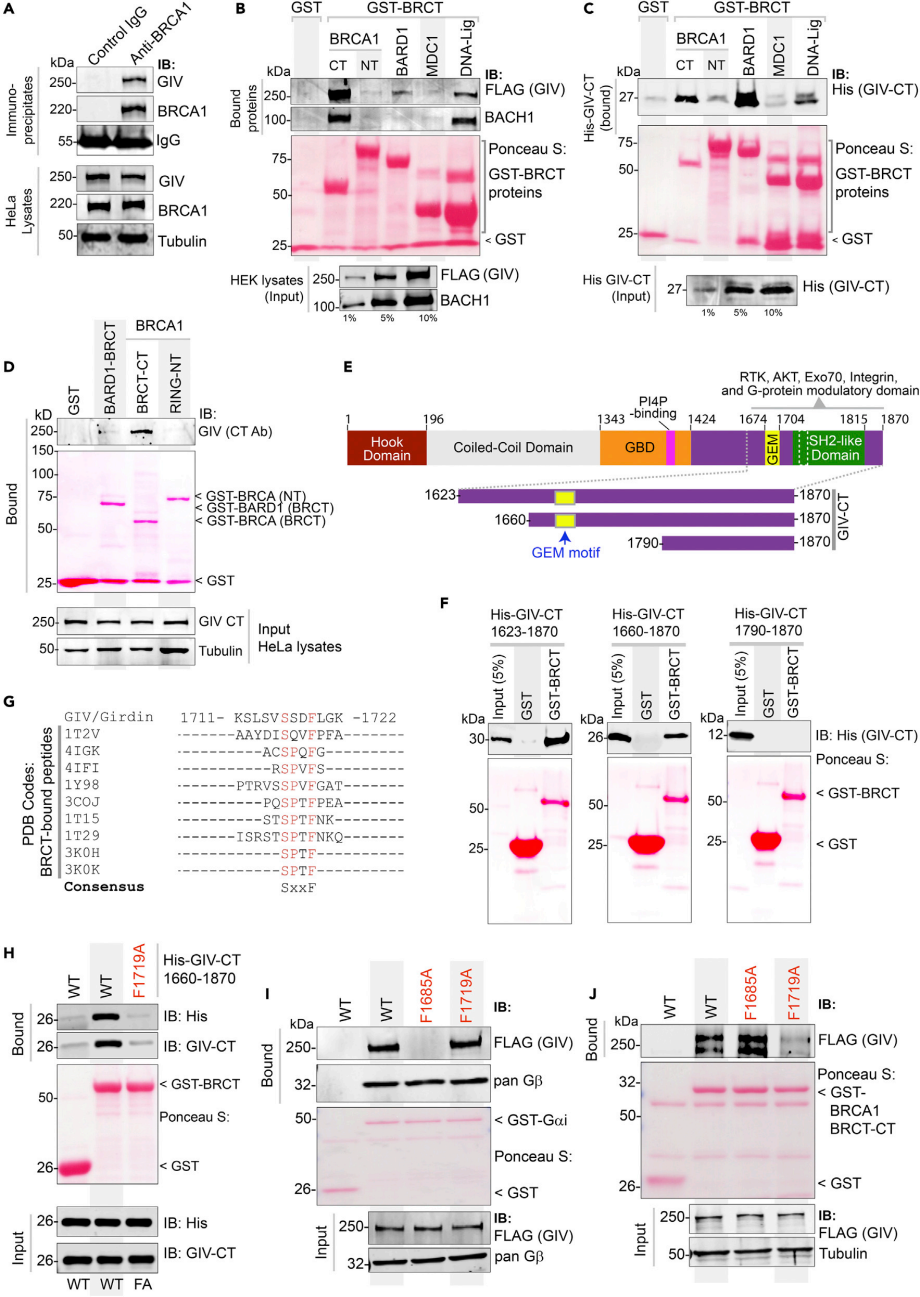
GIV directly binds the BRCT module of BRCA1 (A) Coimmunoprecipitation assays were carried out on lysates of HeLa cells using anti-BRCA1 antibody or control IgG and immune complexes (top) and lysates (bottom) were analyzed for GIV and BRCA1 by immunoblotting. (B) Lysates of HEK cells exogenously expressing FLAG-tagged full-length GIV were used as the source of GIV and endogenous BACH1 (positive control for known BRCA1-binding protein in the same lysates) in pulldown assays with GST-tagged BRCA1 fragments and BRCT modules of various indicated proteins (visualized using Ponceau S). Bound proteins (top) and lysates (bottom) were analyzed for GIV and BACH1. (C) Pulldown assays were carried out using recombinant His-GIV-CT (aa 1660–1870) and GST-BRCT modules as in B. Bound GIV was visualized by immunoblotting (anti-His). (D) Pulldown assays were carried out using lysates of HeLa cells as the source of endogenous full-length GIV with GST-tagged BRCT modules of BRCA1 and BARD1. Bound GIV was visualized by immunoblotting. See also Figure S3 for similar studies with Cos7 and Hs578T cell lysates. (E and F) Recombinant GIV-CT proteins of various lengths (see schematic E) were used in pulldown assays with the GST-BRCT module of BRCA1. Bound GIV-CT fragments were analyzed in F by immunoblotting (His). (G) Alignment of GIV’s C-terminal sequence with known phosphopeptides that bind BRCA1, as confirmed by X-ray crystallography (PDB codes on the left). The consensus SxxF sequence is shown (evolutionary conservation of the SxxF motif and its relationship with other motifs on GIV-CT is shown in Figure S4). (H) Pulldown assays were carried out using His-GIV-CT WT or F1719A mutant with GST/GST-BRCA1 and bound GIV was analyzed by immunoblotting. (I and J) Pulldown assays were carried out with either GDP-loaded GST-Gαi3 (I) or GST-BRCA1 (BRCT; J) proteins and lysates of HEK cells exogenously expressing FLAG-tagged GIV wild-type (WT) or GIV mutants that do not bind G protein (F1685A)^20^ or do not bind BRCA1 (F1719A; current work). Bound proteins were visualized by immunoblotting using anti-FLAG IgG.

We next sought to validate the major BioID-predicted interaction of GIV, i.e., BRCA1. To determine if GIV and BRCA1 interact in cells, we carried out coimmunoprecipitation (Co-IP) assays and found that the two full-length endogenous proteins exist in the same immune complexes (Figure 3A). BRCA1 features two prominent modules that mediate protein-protein interactions, an N-terminal RING domain, which functions as an E3 ubiquitin ligase,^38^ and a C-terminal BRCT repeat domain, which functions as the phospho-protein binding module.^39^ Pulldown assays using recombinant GST-tagged BRCA1-NT (RING) or CT (tandem BRCT repeats) proteins immobilized on Glutathione beads and lysates of HEK cells as a source of FLAG-tagged GIV showed that full-length GIV binds BRCT, but not the RING module (Figure 3B). We noted that GIV is predicted to also interact with other BRCT-domain containing DDR pathway proteins, e.g., DNA Ligase IV (LIG4) and Mediator Of DNA Damage Checkpoint 1 (MDC1) [Human cell map, cell-map.org; a database of BioID proximity map of the HEK293 proteome; accessed on 01/06/2020] and with BARD1 [BioGRID, thebiogrid.org; accessed 09/05/2020]. Pulldown assays with these BRCT modules showed that GIV bound DNA Ligase and BARD1, but not MDC1 (Figure 3B), suggesting that while GIV can promiscuously bind multiple DDR pathway proteins that contain the BRCT module, there may be a basis for selectivity within such apparent promiscuity. As a positive control for BRCT-binding protein, we tracked by immunoblotting the binding of BACH1 from the same lysates, which bound BRCA1’s tandem BRCT module, as expected,^40^ and to a lesser extent with DNA Ligase (Figure 3B).

We next asked if the C-terminus of GIV can directly bind BRCA1; we focused on GIV’s C-terminus (GIV-CT) because numerous studies have underscored the importance of GIV-CT as an unstructured and/or intrinsically disordered domain that scaffolds key proteins within major signaling cascades to mediate dynamic pathway crosstalk.^41^^,^^42^ GST pulldown assays using recombinant His-tagged GIV-CT^1660-1870^ and various GST-DDR pathway proteins showed that GIV’s CT is sufficient to bind the C-terminal tandem BRCT domain of BRCA1 (Figure 3C). Because we used purified recombinant proteins in this assay, we conclude the GIV⋅BRCA1 interaction observed in cells is direct. Using lysates from multiple different cell types as a source of GIV (Hs578T, Figure 3D; Cos7 and HeLa; Figures S3A and S3B) we further confirmed that endogenous full-length GIV binds the C-terminal tandem BRCT domain of BRCA1 (but not its RING domain) and weakly with BARD1.

Domain-mapping efforts, using various fragments of GIV-CT (aa 1623–1870, 1660–1870, and 1790–1870; Figure 3E) helped narrow the region within GIV that binds BRCA1. The longer GIV-CT fragments bound, but the shortest fragment (1790–1870) did not (Figures 3E and 3F), indicating that the sequence of GIV that lies between aa 1660–1790 could be the key determinant of binding. A sequence alignment of this region on GIV against known interactors of BRCA1’s tandem BRCT repeats revealed the presence of a canonical BRCT-binding phospho-peptide sequence of the consensus “phosphoserine (pSer/pS)-x-x-Phenylalanine (Phe; F)” (Figure 3G). The structural basis for such binding has been resolved.^43^ This newly identified putative BRCT-binding motif in GIV had three notable features: First, this motif (^1716^SSDF^1719^) is distinct from and farther downstream of GIV’s Gαi-modulatory motif (31 aa ∼1670–1690) (Figure S4A), suggesting that they may be functionally independent. Second, the SxxF motif is evolutionarily conserved in higher vertebrates (birds and mammals) (Figure S4A), suggesting that GIV could be a part of the complex regulatory capacities that evolved later.^44^ Third, multiple independent studies have reported that the Ser in ^1716^SSDF^1719^ is phosphorylated (Figure S4B), suggesting that GIV⋅BRCA1 complexes may be subject to phosphomodulation. Site-directed mutagenesis that destroys the consensus motif (by replacing Phe with Ala; F1719A) resulted in a loss of binding between GIV-CT and BRCA1 (Figure 3H), thereby confirming that the putative BRCT-binding motif is functional and implicating it in the GIV⋅BRCA1 interaction. The independent nature of the BRCA1-binding and Gαi-modulatory motif was confirmed in pulldown assays with full-length WT and mutant GIV proteins (Figures 3I and 3J); the BRCA1 binding-deficient F1719A mutant protein selectively lost binding to GST-BRCA1, but not GST-Gαi3, and the well-characterized G-protein binding-deficient F1685A mutant protein^20^^,^^21^ selectively lost binding to GST-Gαi3, but not GST-BRCA1.

Collectively, these findings demonstrate that GIV binds BRCA1 via its C-terminally located BRCT-binding motif. This motif is sensitive to disruption via a single point mutation but specific enough that such mutation does not alter GIV’s ability to bind Gαi-proteins.

### GIV binds BRCA1 in both phospho-dependent and -independent modes via the same motif

**Figure 4:**
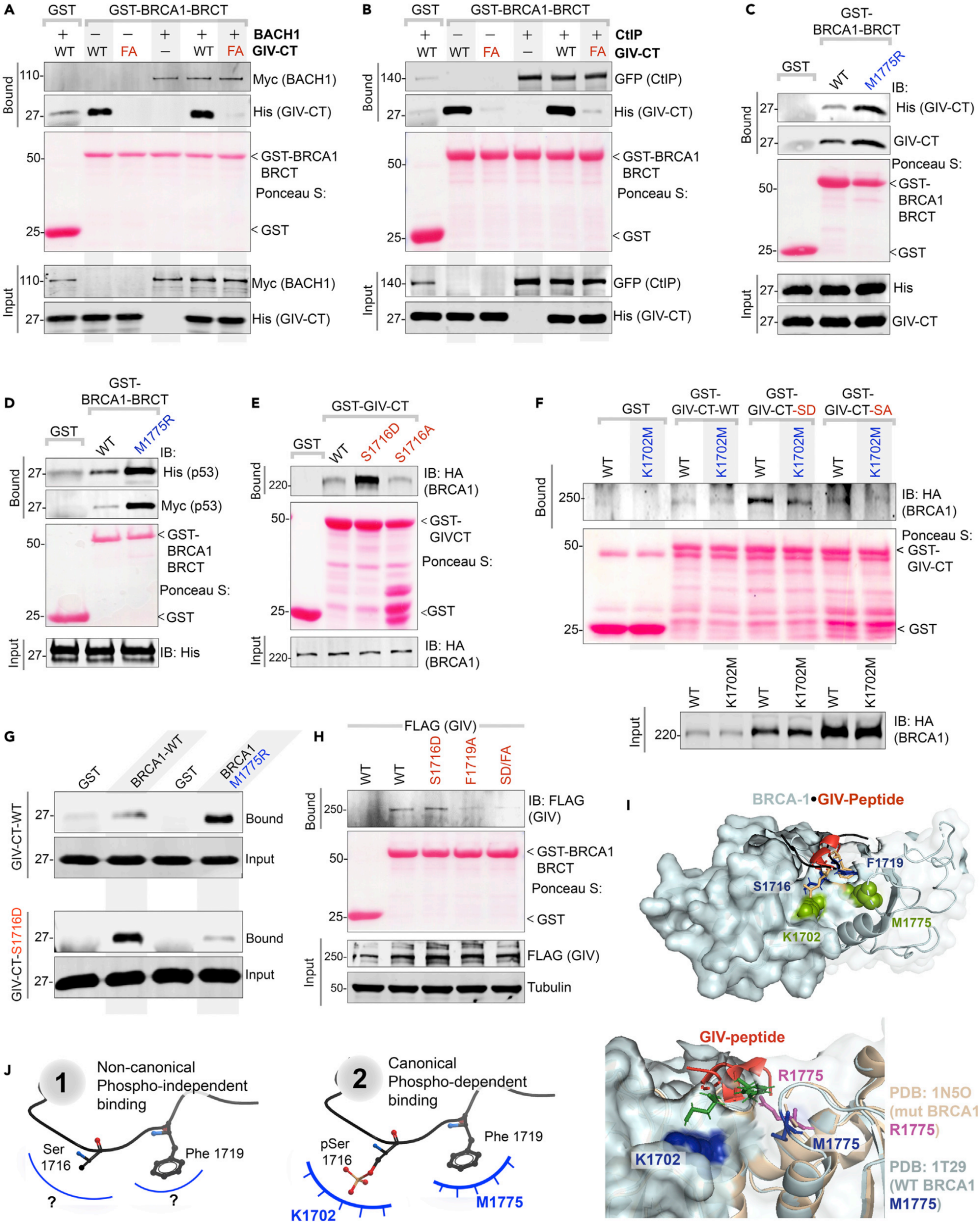
GIV binds BRCA1 via both canonical (phosphodependent) and non-canonical (phosphoindependent) mechanisms (A and B) Binding of unphosphorylated GIV with BRCA1 does not compete with canonical, phospho-dependent binding of BACH1 (A) or CtIP (B). Pulldown assays were carried out using lysates of HEK cells as a source of myc-BACH1 (A) or GFP-CtIP (B) and recombinant GST/GST-BRCA1 proteins, in the presence (+) or absence (−) of either wild-type (WT) or BRCA-binding deficient F1719A (FA) mutant His-GIV-CT at 50-fold molar excess of GST-BRCA1. Bound proteins were visualized by immunoblotting with anti-His (GIV), anti-myc (BACH1; A), or anti-GFP (CtIP; B) IgGs. See also Figure S5. (C and D) Pulldown assays were carried out using His-GIV-CT (C) or His-TP53 (D) and GST or GST-BRCA1 (WT and M1775R mutants). Bound proteins were visualized by immunoblotting with anti-His IgG. (E) Pulldown assays were carried out using lysates of HEK cells as the source of HA-BRCA1 (full length) with either GST (control) or wild-type (WT) and phosphomimic (S1716D) or non-phosphorylatable (S1716A) mutant GST-GIV-CT. Bound BRCA1 was visualized by immunoblotting. (F) Pulldown assays were carried out as in E, using lysates of HEK cells exogenously expressing either wild-type (WT) or K1702M mutant of HA-BRCA1. (G) Pulldown assays were carried out using recombinant His-GIV-CT (WT or S1716D) and either GST-BRCA1 WT or M1775R mutant protein as in C. Bound GIV was visualized by immunoblotting using anti-His IgG. (H) Lysates of HEK cells exogenously expressing full-length GIV-FLAG constructs were used as the source of GIV in pulldown assays with GST/GST-BRCA1. Bound GIV was visualized using anti-FLAG IgG. (I) Homology model of phospho-dependent GIV⋅BRCA1 complex (I; *top*) built using the solved crystal structure of BACH1⋅BRCA1 complex (PDB: IT29) as a template. GIV = red; major residues on BRCA1 or GIV that were mutated here are labeled. Impact of M1775R mutant BRCA1 posing a steric clash with F1719 (GIV) is highlighted (I; *bottom*). (J) Schematic summarizing the two modes of binding of the same ^1716^SxxF^1719^ sequence on GIV-CT to the BRCT module of BRCA1. The structural basis for phospho-independent binding remains unknown (*left*; “?”).

We next asked how GIV binds BRCA1(BRCT). BRCT modules are known to bind ligands via two modes—(i) canonical, phospho-dependent (e.g., BACH1, CtIP, Abraxas) and (ii) non-canonical, phospho-independent (e.g., p53);^45^ while the structural basis for the former has been resolved,^43^ the latter remains unclear. Because bacterially expressed His-GIV-CT directly binds the tandem BRCA1-BRCT (Figure 3C), the GIV⋅BRCA1 (BRCT) interaction appears phospho-independent. As positive controls for canonical phospho-dependent binding, we used BACH1 and CtIP, two *bona fide* binding partners of the BRCA1-BRCT module. Recombinant His-GIV-CT did not impact the canonical mode of binding of either BACH1 (Figure 4A) or CtIP (Figure 4B) to BRCA1-BRCT, suggesting that unphosphorylated GIV binds BRCA1 at a site that is distinct from the interdomain cleft where BACH1 or CtIP are known to occupy.^43^ Furthermore, binding of GIV to the tandem BRCT was enhanced ∼3- to 5-fold in the presence of the most frequently occurring mutation in BRCA1, M1775R (Figure 4C); this mutation is known to abrogate canonical mode of phosphopeptide binding by destroying a hydrophobic pocket that otherwise accommodates the Phe in the pSxxF consensus (see Figure S5A).^46^ The unexpected increase in binding to the BRCA1-M1775R mutant was also observed in the case of p53, which is another direct and phospho-independent BRCA1(BRCT)-interacting partner^47^^,^^48^^,^^49^^,^^50^ (Figure 4D). The expected disruptive effect of this mutation could, however, be confirmed in the case of both BACH1 (Figure S5B) and CtIP (Figure S5C). These findings demonstrate that GIV binds BRCA1 via a non-canonical phospho-independent mechanism that is distinct from CtIP and BACH1.

Because ∼10 high-throughput (HTP) studies have confirmed that Ser^1716^ within the BRCA1-binding motif of GIV is phosphorylated (Figure S5C), presumably by one of the many DDR and cell-cycle regulatory kinases (Figure S5D), we asked if the GIV⋅BRCA1 interaction is phosphomodulated. Phosphomimic (Ser^1716^→Asp; S1716D) and non-phosphorylatable (Ser^1716^→Ala; S1716A) mutants of GST-GIV-CT were generated, rationalized based on systematic peptide screening studies demonstrating that Glu/Asp-x-x-Phe peptides bind BRCT modules with ∼10-fold higher affinity.^51^ Binding of BRCA1 was accentuated with GIV-S1716D mutant but restored to levels similar to WT in the case of GIV-S1716A mutant (Figure 4E), indicating that the GIV⋅BRCA1 interaction may be phosphoenhanced and that the -OH group in Ser (which is absent in Ala; A) is not essential for the interaction. The phosphate group in the consensus pSxxF mediates polar interactions with S1655/G1656 in β1 and K1702 in α2 of BRCA1,^43^ and a K1702M mutant has previously been shown to impair phospho-dependent canonical mode of binding.^44^ We found that the observed phosphoenhanced GIV⋅BRCA1 in Figure 4E is virtually abrogated in the case of BRCA1-K1702M (Figure 4F), indicating that upon phosphorylation at S1716, GIV may bind BRCA1 in a phospho-dependent canonical mode. Finally, in pulldown assays with the BRCA1-M1775R mutant, binding was inhibited to the phosphomimic GIV-S1716D mutant, but not to GIV-WT (Figure 4G), likely via the obliteration of the binding pocket for the F1719, as has been reported in the canonical binding mode.^46^ That the F1719 is also important for phospho-dependent binding was also confirmed; the addition of F1719A mutation to S1716D mutation disrupted binding to BRCA1 (Figure 4H), indicating that the same BRCA1-binding motif participates in both modes of binding. Homology models of GIV⋅BRCA1 co-complexes (Figure 4I; *top*), built using the solved structure of canonical BACH1⋅BRCA1 co-complex (PDB:1T29) as template further confirmed that phospho-dependent canonical mode of binding and disrupted binding when M1775 is mutated to R (Figure 4I; *bottom*) is compatible with the observed biochemical studies.

Taken together, these findings support the conclusion that GIV binds BRCA1 in two different modes: a non-canonical phospho-independent mode, the structural basis for which remains unknown (Figure 4J; *left*), and a canonical phospho-dependent mode (Figure 4J; *right*). Both modes of binding occur via the same motif in GIV.

### Both GIV⋅BRCA1 and GIV⋅Gαi interactions are required for DNA repair

**Figure 5:**
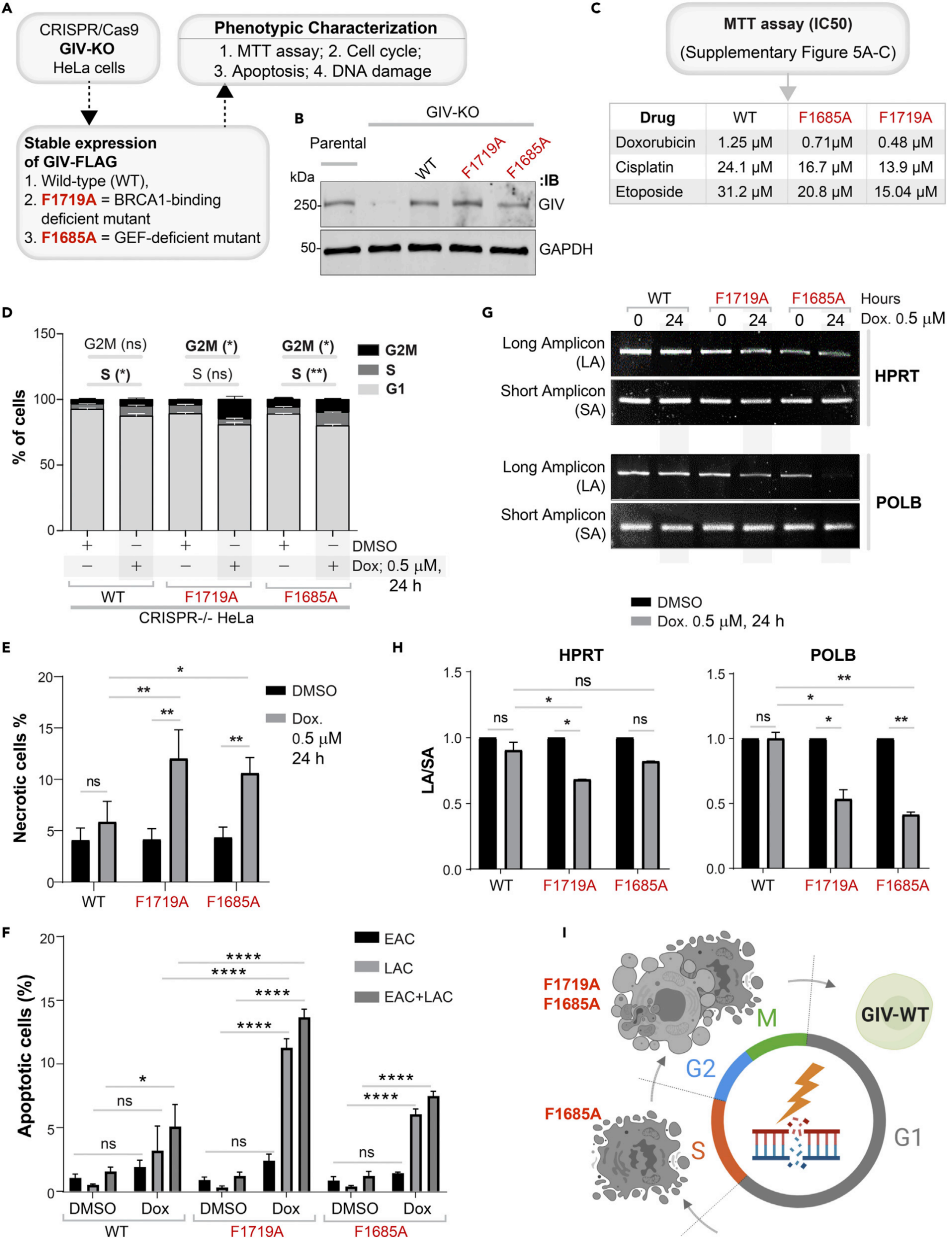
DNA damage repair response is impaired in cells expressing mutant GIV that cannot bind BRCA1 (F1719A) or bind/activate G proteins (F1685A) (A) Schematic outlining the cell lines and phenotypic assays displayed in this figure. (B) Immunoblot of the HeLa cell lysates showing the depletion of full-length endogenous GIV, followed by rescue WT and mutant GIV at levels close to endogenous. (C) Table of IC50 values for 3 different drugs tested on GIV-WT, GIV-F1685A, and GIV-F1719A HeLa cells, as determined using MTT assays. See Figures S6A–S6D for the dose-dependent survival curves. (D) Stacked bar graphs showing the percentage of cells at various stages of the cell cycle (G1, S, and G2/M) after challenged with Dox or vehicle control (DMSO). Data displayed as mean ± SEM and one-way ANOVA using Tukey’s multiple comparisons test was used to determine significance. (∗; p ≤ 0.05, ∗∗; p ≤ 0.01; ns = not significant). Histograms are shown in Figure S6E. (E and F) Bar graphs display the % necrotic (E) or apoptotic (early, EAC; late, LAC; or combined) cells after challenge with either Dox or vehicle control (DMSO) as assessed by annexin V staining and flow cytometry. See Figure S6F for the dot plot diagrams. (G and H) Long amplicon qPCR (LA-QPCR) was used to evaluate genomic DNA SB levels in various HeLa cell lines. Representative gel showing PCR-amplified fragments of the *HPRT* (G, top panel) and *POLB* (G, bottom panel) genes. Amplification of each large fragment (upper panels) was normalized to that of a small fragment of the corresponding gene (bottom panels) and the data were expressed as normalized (with short PCR amplicon) relative band intensity with the DMSO-treated (0 h) sample in each case arbitrarily set as unity and displayed as a bar graph in H. Full-length gels can be seen in Figure S6G. Data displayed as mean ± SEM and one-way ANOVA to determine significance. (∗; p ≤ 0.05; ∗∗∗∗; p ≤ 0.0001; ns = not significant). (I) Schematic summarizing the findings in cells with GIV-WT or mutants that either cannot bind G protein (F1685A) or BRCA1 (F1719A). See also Table S3 for a summary of all phenotypes observed in these mutants.

To dissect the role of the GIV⋅BRCA1 interaction, we rescued GIV KO HeLa cell lines with either GIV-WT or single-point specific mutants of GIV that either cannot bind BRCA1 (F1719A) or cannot bind/activate Gαi-proteins (F1685A) and used them in the same phenotypic assays as before (Figure 5A). First, we confirmed by immunoblotting that the G418-selected clones stably express physiologic amounts of GIV-WT/mutants at levels similar to endogenous (Figure 5B). When challenged with Dox, cisplatin, or etoposide, survival, as determined using an MTT assay was significantly reduced in the cells expressing either mutant compared to GIV-WT (Figures 5C and S6A–S6C). The lower IC50 values in the case of GIV mutant cell lines for all 3 drugs (Figure 5C) imply that both functions of GIV, i.e., BRCA1-binding and G protein-binding/activating, are required for surviving cytotoxic lesions induced by commonly used cytotoxic drugs. Lower survival was associated with G2/M phase arrest in both mutant lines (Figures 5D and S6A). The S phase checkpoint, however, was intact in cells expressing GIV-WT and GIV-F1685A mutant, but not in GIV-F1719A mutant (Figure 5D), indicating that the disruption of the GIV⋅BRCA1 interaction blocks the S phase checkpoint. Flow cytometry studies showed that cell death, both necrosis (Figures 5E and S6B) and apoptosis (late apoptosis; LAC; Figures 5F and S6B), was significantly increased in both mutant-expressing lines compared to GIV-WT. The extent of death was higher in GIV-F1719A mutant lines, indicating that the disruption of the GIV⋅BRCA1 interaction is catastrophic. Consistently, the burden of mutations was increased in both mutant lines, but to a higher degree in GIV-F1719A mutant lines (Figures 5G, 5H, and S6C).

Taken together, these results demonstrate that both functions of GIV (BRCA1-binding and Gαi binding and activation) are important for GIV’s role in mounting a DDR. The use of GIV KO cell lines rescued with WT or specific binding-deficient mutants further pinpointed the role of each function in the process (Figure 5I; summarized in Table S3). The GIV⋅BRCA1 interaction was required for S phase checkpoint arrest, cell survival, and DNA repair. However, GIV’s Gαi-modulatory function was somehow important for cell survival and the efficiency of DNA repair.

### GIV may inhibit homologous recombination and favor non-homologous end-joining

**Figure 6:**
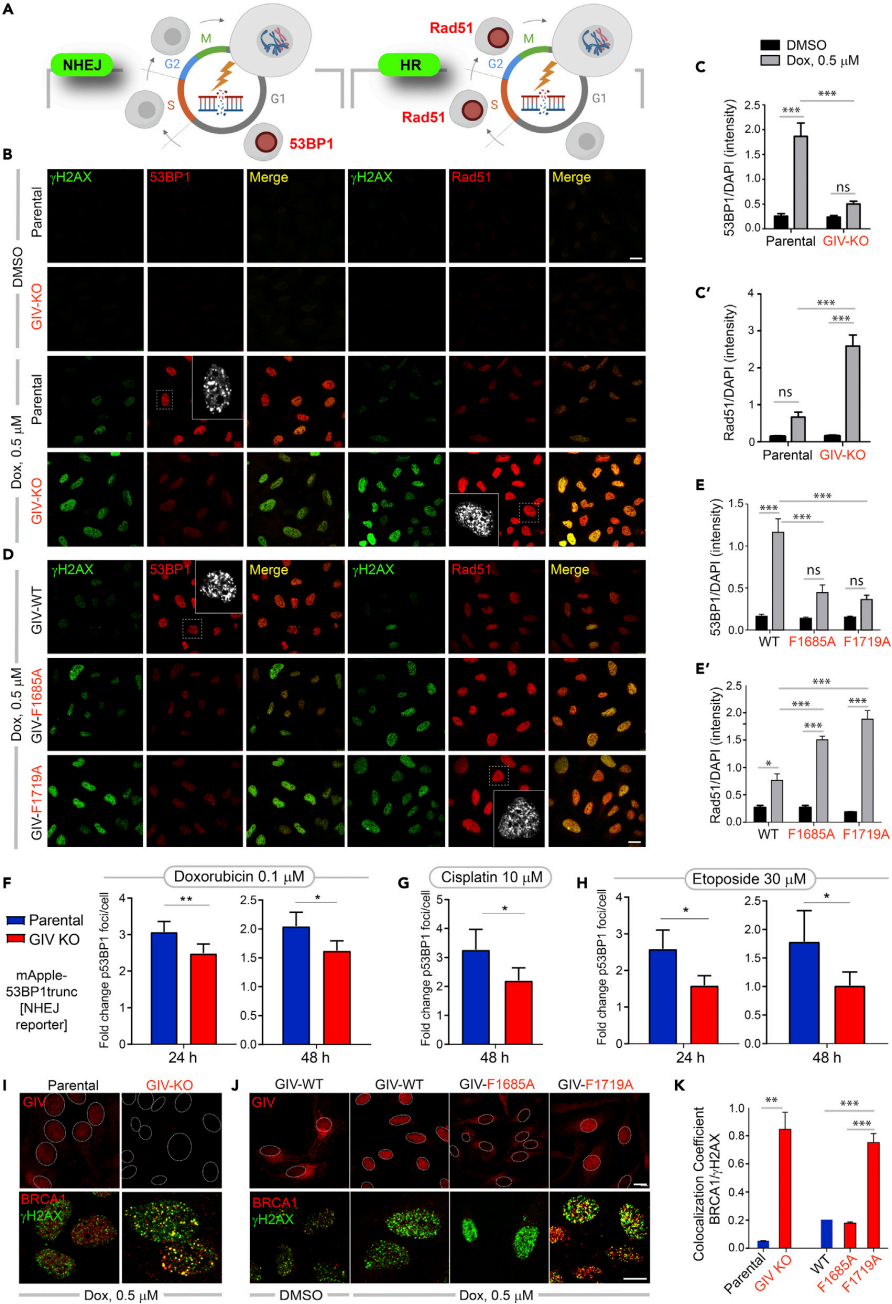
GIV inhibits HR, favors NHEJ, and inhibits the localization of BRCA1 to sites of DNA damage (A) Schematic summarizing the two markers, 53BP1 (left) and Rad51 (right) commonly used to monitor the repair pathway of choice (NHEJ vs. HR, respectively) after DNA damage. (B–E′) Control (parental) and GIV-depleted (GIV KO) HeLa cells (B-C) or GIV-depleted HeLa cells stably expressing WT or mutant GIV constructs (D-E) were challenged with Dox or vehicle control (DMSO) prior to being fixed with Methanol and co-stained for γH2AX (green) and 53BP1 (red; left) or Rad51 (red; right) and analyzed by confocal microscopy. Representative images are shown in B and D (scale bar = 15 μm). Insets show the magnified view of a single cell (interrupted box) in the field, highlighting the punctate nature of the nuclear staining for 53BP1 or Rad51. Bar graphs in C-C′ and E-E′ show the quantification of the intensity of 53BP1 or Rad51 staining normalized to DAPI. Data displayed as mean ± SEM and one-way ANOVA to determine significance. (∗; p ≤ 0.05; ∗∗; p ≤ 0.01; ∗∗∗; p ≤ 0.001; ns = not significant). (F–H) Bar graphs display the fold change in the number of bright foci of 53BP1 in parental and GIV KO HeLa cells stably expressing mApple-53BP1 reporter (which detects NHEJ) upon challenge with the indicated concentrations of Doxorubicin (F), Cisplatin (G) or Etoposide (H). Data displayed as mean ± SEM and t-test to determine significance. (∗; p ≤ 0.05; ∗∗; p ≤ 0.01). See also Figures S7A and S7B for 53BP1 reporter studies on parental and GIV KO MDA-MB-231cells. (I–K) HeLa cell lines in B, D were treated as in B, D, and fixed and analyzed for GIV (top) and BRCA1 (bottom) localization with respect to the nuclei (demarcated with interrupted oval outlines). Representative images are shown in I-J (scale bar = 15 μm). See Table S4 for predicted nuclear localization signals in GIV. See also Figures S7B and S7C for expanded individual panels. Bar graphs in K show Pearson’s colocalization coefficient for the degree of colocalization observed within the nucleus between BRCA1 (red) and γH2AX (green).

We next asked how GIV’s ability to bind BRCA1 or activate Gαi might impact the choice of the pathway for DNA repair. While γH2AX is responsible for the recruitment of many DNA maintenance and repair proteins to the damaged sites, including 53BP1 and RAD51,^52^ the preferential accumulation of 53BP1 indicates NHEJ, whereas the preferential accumulation of Rad51 indicates HR^12^ (Figure 6A). BRCA1 favorably activates Rad51-mediated HR repair and actively inhibits 53BP1-mediated NHEJ repair.^53^ We found that the nuclear accumulation of 53BP1, as determined by confocal microscopy, was higher in parental HeLa cells compared to GIV KO cells (Figures 6B; *left*; 6C). By contrast, nuclear accumulation of Rad51 was much more pronounced in GIV KO compared to parental cells (Figures 6B; *right*; 6C′). These findings indicate that NHEJ is the preferred choice for repair in cells with GIV, but HR is favored in the absence of GIV. This preference of HR over NHEJ in GIV KO cells was reversed in KO cells rescued with GIV-WT but could not be rescued by mutant GIV proteins that could not bind BRCA1 or modulate Gαi proteins (Figures 6D and 6E-6E′). DSBs were increased in GIV KO cells and in cells expressing either of the GIV mutants, as determined by γH2AX staining; this is consistent with the prior long amplicon PCR studies assessing the burden of mutations (Figures 2G, 2H, 5G, and 5H).

That GIV is required for NHEJ was further confirmed by live cell imaging using parental and GIV KO HeLa (Figures 6F–6H) and MDA-MB-231cells (Figures S7A and S7B) stably expressing a fluorescent reporter of endogenous DSBs, a construct comprised of a truncated segment of p53BP1 fused to mApple^54^ (see STAR Methods *for details*). Compared to the parental cells, the fold change in bright foci/cell was significantly decreased in GIV-depleted HeLa and MDA-MB-231cells regardless of the drugs, duration and concentrations tested.

Taken together, these findings suggest that GIV and its BRCA1-binding and Gαi-modulatory functional modules may influence the choice of DDR; when GIV is present and its two functional modules are intact, 53BP1 is preferentially recruited to DSBs, in the detriment of Rad51, indicating that NHEJ may be preferred over HR. It is also noteworthy that the mutational burden is increased despite the DNA damage-induced accumulation of nuclear Rad51, which suggests that HR is initiated successfully, but may not be as effective as NHEJ. The latter offers an ideal balance of flexibility and accuracy in the setting of widespread DSBs with diverse end structures.^15^

### GIV translocates to the nucleus after DNA damage, inhibits the colocalization of BRCA1 with double-strand breaks

Because BRCA1 is a nucleocytoplasmic shuttling protein^55^ and it is nuclear BRCA1 that augments DNA repair^56^ and cell-cycle checkpoints,^57^ we asked if suppressed HR in cells with GIV, or those with functionally intact modules in GIV stemmed from the mis-localization of BRCA1. We determined the localization of GIV and BRCA1 by confocal immunofluorescence and found that the Dox challenge was associated with the nuclear localization of GIV (see Parental cells; Figure 6I, *top-left*). Compared to parental control cells, nuclear localization of BRCA1 was more prominent in GIV KO cells (Figure S7C), where BRCA1 colocalized with γH2AX (see Figure 6I, *bottom*; *see* Figure 6K for colocalization index), indicating that the nuclear localization of BRCA1 to sites of DSBs may be suppressed by GIV.

To discern which functional module of GIV may be important for the nuclear localization of GIV and/or suppression of the nuclear localization of BRCA1, we carried out similar assays in stable cell lines expressing GIV-WT or mutant. DNA damage-dependent shuttling of GIV to the nucleus was observed in the case of GIV-WT and GIV-F1719A, but not GIV-F1685A (see Figure 6J, *top*), indicating that GIV’s ability to shuttle into the nucleus after DNA damage does not depend on its interaction with BRCA1, but requires a functionally intact Gαi-modulatory function. We observed prominent nuclear localization of BRCA1 only in the GIV-F1719A mutant line (Figure S7D), where it colocalized with γH2AX (see Figure 6J, *bottom*). Colocalization coefficient of BRCA1 with γH2AX across all cell lines showed that colocalization was greatest in the absence of GIV (GIV KO cells; Figures 6K and S7C) or when the GIV⋅BRCA1 interaction is impaired (F1719A; Figure S7D), indicating that the GIV⋅BRCA1 interaction is required for the observed inhibitory effect of GIV on the nuclear localization of BRCA1. Nuclear-cytosol fractionation studies also showed that the nuclear pool of BRCA1 and Rad51 in Dox-challenged HeLa cells is increased in the absence of GIV (Figure S7E).

Taken together, these findings demonstrate that GIV, like BRCA1, is a nucleocytoplasmic shuttling protein; shuttling is independent of its BRCA1-binding function but depends on its Gαi-modulatory function. The GIV⋅BRCA1 interaction appears to be primarily responsible for sequestering BRCA1 away from DSBs. Localization of BRCA1 at sites of DSBs is not only impaired in the case of GIV-WT expressing cells, in which GIV shuttles into the nucleus upon DNA damage, but also impaired in GIV-F1685A mutant cells (Figures 6H and S7D), in which GIV fails to localize to the nucleus. This indicates that the inhibitory GIV⋅BRCA1 interaction may occur in the nucleus as well as in the cytoplasm, and is in keeping with our BioID studies revealing BRCA1 as a candidate interactor of GIV in both nuclear and cytosolic compartments (Figure 1C).

### GIV’s Gαi-modulatory function activates Akt, BRCA1-binding function triggers S-phase checkpoint

**Figure 7:**
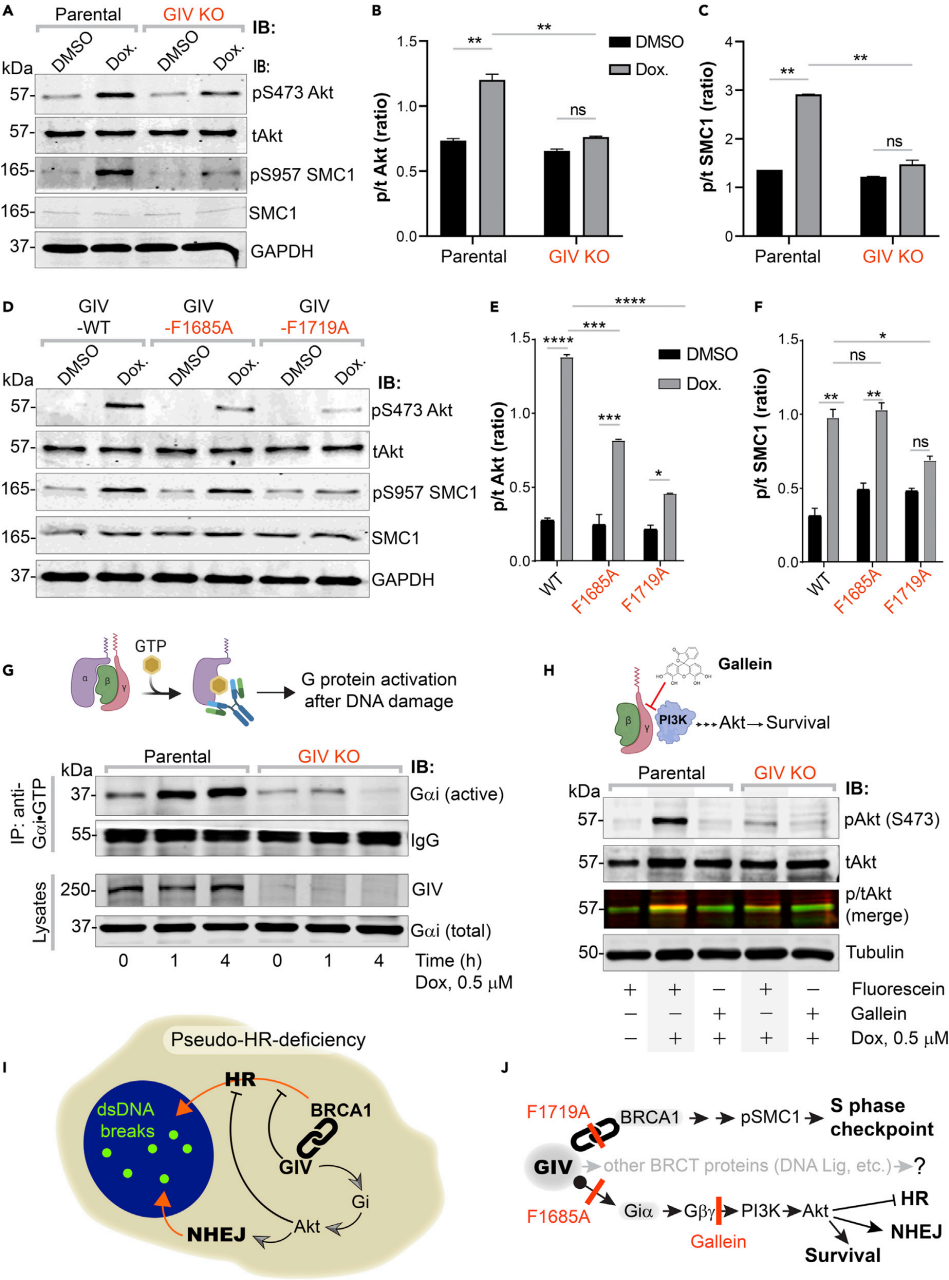
Activation of Gi by GIV is required for Akt enhancement during DDR, contributes to pseudo-HR-deficiency (A–F) Control (parental) and GIV-depleted (GIV KO) HeLa cells (A-C) or GIV-depleted cells stably expressing WT or mutant GIV constructs (D-F) were challenged with Dox or vehicle control (DMSO) as indicated prior to lysis. Equal aliquots of lysates were analyzed for total (t) and phosphorylated (p) Akt and SMC1 proteins and GAPDH (loading control) by quantitative immunoblotting using LiCOR Odyssey. Representative immunoblots are shown in A and D, and quantification of phospho(p)/total(t) proteins is displayed as bar graphs in B, C, E, F. Data displayed as mean ± SEM and one-way ANOVA to determine significance. (∗; p ≤ 0.05; ∗∗; p ≤ 0.01; ∗∗∗; p ≤ 0.001; ∗∗∗∗; p ≤ 0.0001; ns = not significant). (G) Schematic on top shows the assay used for assessing the extent of Gαi-activation using conformation-sensitive antibodies that selectively bind the GTP-bound (active) conformation of Gαi protein. Immunoblots below show the active Gαi immunoprecipitated (top; IP) from lysates (bottom) of HeLa cells treated with Dox. for the indicated time points. (H) GIV-depleted (GIV KO) and control (Parental) HeLa cells were stimulated (+) or not (−) with Dox. as indicated, in the presence of either Gallein or its inactive isomer, Fluorescein. Equal aliquots of lysates were immunoblotted for pAkt and tAkt as in panel A. (I and J) Summary of findings showing how GIV skews the choice of repair pathway from HR to NHEJ, partly via sequestration of BRCA1 away from the sites of dsDNA breaks and in part via the enhancement of Akt via the Gi→“free” Gβγ→Class I PI3K pathway. The tools (mutants and chemical inhibitors) used in this work are highlighted in red.

Because the choice of DNA damage repair pathway is fine-tuned by a network of kinases (e.g., ATM, ATR, Akt, and so forth) and the signaling cascades they initiate,^58^^,^^59^ we asked how GIV and its functional modules may impact these pathways. More specifically, we focused on two key readouts rationalized by our observations: (i) Akt phosphorylation, because GIV is a *bona fide* enhancer of Akt phosphorylation^60^^,^^61^ and does so via its Gαi-modulatory function,^20^ and because this pathway is known to impact the choice of repair [reviewed in^59^]; (ii) Phosphorylation of the cohesion protein, Structural Maintenance of Chromosome-1 (pSer-957 SMC1),^62^ a readout of S-phase checkpoint, because this checkpoint was impaired in GIV KO cells (Figure 2D). We found that the depletion of GIV significantly reduced phosphorylation of both readouts (Figures 7A–7C), indicating that GIV is required for the phosphoactivation of both the Akt and the ATM→pSMC1 axes. Similar studies on HeLa cell lines stably expressing GIV-WT or mutants showed that Akt phosphrylation was impaired in both GIV-F1685A and GIV-F1719A mutants (Figures 7D and 7E), albeit more significantly impaired in the latter, but phosphoSMC1 was specifically impaired in cells expressing the GIV-F1719A mutant (Figures 7D and 7F). These findings show that the BRCA1-binding function of GIV is critical for the initiation of Akt signaling upon DNA damage, as well as for the activation of the ATM→pSMC1 pathway for S-phase checkpoint signaling. The Gαi-modulatory function of GIV, however, was specifically responsible for enhancing Akt signals after DNA damage.

We asked if the previously delineated Gi → “free” Gβγ release → Class 1 PI3K signaling axis triggered by GIV’s Gαi-modulatory function may be essential.^20^ To this end, we first assessed the extent of the activation of Gαi in cells after DNA damage by using a conformation-sensing antibody that specifically recognizes GTP-bound (active) conformation of Gαi1-3 (Figure 7G; *top*),^63^ and more importantly, recognize GIV-dependent G protein activation in cells.^42^ We found that DNA damage was associated with the activation of Gαi in parental cells, but that such activation was virtually lost in GIV KO cells (Figure 7G; *bottom*). To dissect if Akt activation is mediated via the “free” Gβγ→Class 1 PI3K signaling axis, we used the commonly used small molecule Gβγ inhibitor, Gallein (Figure 7H; *top*), and it’s an inactive isomer, Fluorescein (negative control).^64^ We found that Gallein, but not Fluorescein inhibited DNA damage-induced Akt phosphorylation in parental control cells, reducing it to the levels observed in GIV KO cells (Figure 7H; *bottom*). These findings indicate that Akt signaling induced after DNA damage occurs in part via GIV-dependent Gi activation.

Taken together, our findings support the following working model for how GIV may influence the choice of repair pathway after DNA damage, favoring NHEJ over HR (Figure 7I). Using a set of single-point mutants and chemical inhibitors of G protein signaling, we charted the mechanisms that allow GIV to accomplish such a goal via two parallel pathways (see Figure 7J). One pathway is mediated by GIV’s ability to bind and sequester BRCA1 in the cytoplasmic pool, and thereby reducing its ability to localize to DSBs, suppress HR, and activate S phase checkpoint cascades. Another is GIV’s ability to bind and activate Gi and enhance Akt signaling, which further skews the choice of repair pathway toward NHEJ, while actively suppressing HR.

## Discussion

Cellular decision-making in response to any stressful insult is mediated by a web of spatiotemporally segregated events within the intracellular signaling networks, often requiring crosstalk between unlikely pathways. The major discovery we report here is such an unexpected crosstalk that is orchestrated via a versatile multi-modular signal transducer, GIV/Girdin. There are three notable takeaways from this study.

First, this work ushers a new player in DNA repair. Although GIV entered the field of cancer biology more than a decade ago, and quickly came to be known as a pro-oncogenic protein that coupled G protein signaling with unlikely pathways [reviewed in^24^], its role inside the nucleus remained unknown. Although predicted to have nuclear localization signals (NLS; Table S4), how GIV shuttles into the nucleus remains unresolved. Regardless, what emerged using specific single-point mutants is that GIV inhibits HR by sequestering BRCA1, suppressing its localization to DSBs.

Second, one of the most unexpected observations was that GIV uses the same short linear motif (SLIM) located within its C-terminus to bind the C-terminal tandem-BRCT modules of BRCA1 in both canonical (phospho-dependent) and non-canonical (phospho-independent) modes. Although both modes of BRCT-binding have been recognized in other instances,^45^ the versatility of dual-mode binding via the same motif is unprecedented. However, these findings are in keeping with the fact that GIV-CT is an intrinsically disordered protein (IDP)^41^^,^^65^ comprised of distinct SLIMs, of which the BRCT-binding motif described here is an example (see Figure S3A). SLIMs enable GIV to couple G protein signaling to a myriad of molecular sensors, of both the outside of the cell (i.e., receptors; [reviewed in^66^]) or its interior.^67^ Because IDPs that fold/unfold on demand expose/hide SLIMs, which in turn imparts plasticity to protein-protein interaction networks during signal transduction,^68^ GIV may do something similar in couple G protein signaling to DDR. Given this degree of versatility of the BRCT-binding SLIM in GIV, and the additional BRCT interactors we found here (to DNA Lig IV and BARD1), it is more likely than not that this SLIM binds other players within the DDR pathways. By scaffolding G proteins to BRCT-modules in BRCA1 (and presumably other DDR proteins) GIV may serve as a point of convergence for coordinating signaling events and generating pathway crosstalk upon DNA damage.

Third, this work provides a direct mechanistic link between DDR and trimeric G proteins; the latter is one of the major pervasive signaling hubs in eukaryotic cells that was notably absent from the field of DNA repair. Although multiple peripheral components within the GPCR/G-protein signaling system have been found to indirectly influence DNA damage and/or repair,^23^ who/what might activate G proteins on endomembranes was unknown. We demonstrated that trimeric Gi proteins are activated upon DNA damage and that such activation requires GIV’s Gαi-modulatory motif. That the GIV→Gαi pathway activates Akt signaling helps explain the hitherto elusive origin of Akt signaling during DDR.^59^ That GIV favors NHEJ over HR and activates Akt signaling during DDR is in keeping with the previously described role of Akt signaling in inhibiting HR and promoting NHEJ.^59^^,^^69^^,^^70^^,^^71^^,^^72^

### Limitations of the study

Although how GIV binds BRCA1 was studied at greater depth, how exactly GIV may inhibit the shuttling/localization of BRCA1 remains unresolved. Because nuclear import of BRCA1 and its retention requires BARD1,^73^ whereas nuclear export requires p53,^74^ GIV may either inhibit the BARD1⋅BRCA1 interplay or augment the actions of p53. Although we could gain some insight into the mechanism of GIV⋅BRCA1 interaction, the stoichiometry of this complex in cells remains unknown. Neither do we know the mechanism of how GIV binds DNA Lig IV and BARD1. Because phospho-peptide⋅BRCT interactions are generally believed to be exclusive, GIV’s interaction with DNA Lig IV and BARD1 we observed here are likely to be phospho-independent because they were observed using bacterially expressed recombinant proteins. Which DDR proteins bind GIV, and which do not, may be dictated by the residues flanking the SLIM, as shown in other instances;^75^ additional mutagenesis or structural studies are required to understand the selectivity and specificity of GIV⋅BRCT interactions.

In closing, damage to the genome can have catastrophic consequences, including cytotoxicity, accelerated aging, and predisposition to cancers. Our findings, which revealed a hitherto unknown link between a major hub in DNA repair (i.e., BRCA1) and a signaling hub of paramount importance in just about all aspects of modern medicine (trimeric G proteins) open new avenues for the development of therapeutic strategies.

## STAR★Methods

### Key resources table

**Table:** 

REAGENT or RESOURCE	SOURCE	IDENTIFIER
**Antibodies**		

Mouse monoclonal anti-Myc	Cell Signaling Technology	2276S; RRID:AB_331783
Mouse monoclonal anti-GAPDH	Santa Cruz Biotechnology	sc-365062; RRID:AB_10847862
Rabbit polyclonal anti-BRCA1	Santa Cruz Biotechnology	sc-642; RRID:AB_630944 and sc-6954 (IB, IP); RRID:AB_626761
Mouse monoclonal anti-α-tubulin	Santa Cruz Biotechnology	sc-5286; RRID:AB_628411
Mouse monoclonal anti-FLAG	Millipore Sigma	MAB3118; RRID:AB_94705
Mouse monoclonal anti-GST	GenScript	A00865; RRID:AB_914654
Rabbit polyclonal anti-BACH1	Proteintech	14018-1-AP; RRID:AB_2274498
Rabbit polyclonal anti-pan Gβ	Proteintech	sc-378; RRID:AB_631542
Rabbit polyclonal anti-GFP(CtIP)	Santa Cruz Biotechnology	sc-9996; RRID:AB_627695
Mouse monoclonal anti-p53	Santa Cruz Biotechnology	sc-99; RRID:AB_628086
Mouse monoclonal anti-p-ϒH2A.X	Santa Cruz Biotechnology	sc-517348;RRID:AB_2783871
Rabbit polyclonal anti-53BP1	Cell Signaling Technology	4937S; RRID:AB_10694558
Rabbit polyclonal anti-RAD51	Proteintech	14961-1-AP; RRID:AB_2177083
Rabbit monoclonal anti-pS473 AKT	Cell Signaling Technology	D9E (Clone); Cat# 4060, RRID:AB_2315049
Mouse monoclonal anti-total AKT	Cell Signaling Technology	40D4 (Clone); Cat# 2920, RRID:AB_1147620
Mouse monoclonal anti-pS957 SMC	Cell Signaling Technology	5D11G5 (Clone); Cat# 4805, RRID:AB_2192322
Mouse monoclonal anti-SMC	Cell Signaling Technology	8E6 (Clone); Cat# 6892, RRID:AB_10828353
Rabbit polyclonal anti-Gαi3 (C-10)	Santa Cruz Biotechnology	Cat# sc-262, RRID:AB_2279066
Rabbit polyclonal anti-Gαi (total)	Santa Cruz Biotechnology	sc-389; RRID:AB_2294749
Mouse monoclonal anti- Gαi-GTP	*New East Biosciences and Graeme Milligan*^63^	Cat# 26901, RRID:AB_1961774
Goat anti-Rabbit IgG, Alexa Fluor 594 conjugated	ThermoFisher Scientific	A11072; RRID:AB_2534116
Goat anti-Mouse IgG, Alexa Fluor 488 conjugated	ThermoFisher Scientific	A11017; RRID:AB_2534084
IRDye 800CW Goat anti-Mouse IgG Secondary (1:10,000)	LI-COR Biosciences	926–32210; RRID:AB_621842
IRDye 680RD Goat anti-Rabbit IgG Secondary (1:10,000)	LI-COR Biosciences	926–68071; RRID:AB_10956166

**Chemicals, peptides, and recombinant proteins**		

Biotin	Sigma-Aldrich	B4639-500MG
DAPI (4′,6-Diamidino-2-Phenylindole, Dilactate)	Thermo Fisher Scientific	D3571
G418	Cellgro	A-1720
Paraformaldehyde 16%	Electron Microscopy Biosciences	15710
DIG RNA Labeling Mix	Roche	11277073910
T7 RNA polymerase	Promega	P2075
Doxorubicin	Sigma Aldrich	D1515-10MG
Gallein	TCI Chemicals	2103-64-2
Fluorescein	TCI Chemicals	2321-07-5
MTT	Millipore Sigma	475989-1GM
Cisplatin	EMD Millipore	232120-50MG
Etoposide	Sigma-Aldrich	E1383-25MG
Guava Cell Cycle Reagent	Millipore Sigma	4700-0160
Dead Cell Apoptosis Kit with Annexin V Alexa Fluor™ 488 & Propidium Iodide (PI)	ThermoFisher Scientific	V13241
DAPI (4′,6-Diamidino-2-Phenylindole, Dilactate)	Thermo Fisher Scientific	D3571
Streptavidin, Alexa Fluor® 680 conjugate	ThermoFisher Scientific	S21378
Streptavidin, Alexa Fluor® 594 conjugate	ThermoFisher Scientific	S11227
HisPur^ä^ Cobalt Resin	Thermo Scientific	89964
Glutathione Sepharose^â^ 4B	Sigma-Aldrich	GE17-0756-04
Streptavidin Magnetic Beads	ThermoFisher Scientific	88816
HisPur^ä^ Cobalt Resin	Thermo Scientific	89964
Glutathione Sepharose^â^ 4B	Sigma-Aldrich	GE17-0756-04
Streptavidin Magnetic Beads	ThermoFisher Scientific	88816
Protein A Agarose	ThermoFisher	15918014
Protein G Agarose	ThermoFisher	20398
Protease inhibitor cocktail	Roche	11 873 580 001
Tyr phosphatase inhibitor cocktail	Sigma-Aldrich	P5726
Ser/Thr phosphatase inhibitor cocktail	Sigma-Aldrich	P0044
Paraformaldehyde 16%	Electron Microscopy Biosciences	15710
PVDF Transfer Membrane, 0.45mM	Thermo Scientific	88518

**Deposited data**		

Proteomic dataset analyzed in this work	Ear et al.^37^	PXD022601

**Experimental models: Cell lines**		

HeLa parental	ATCC	ATCC® CCL-2
HeLa GIV KO (CRISPR Cas9)	*This paper*	N/A
HeLa GIV WT	*This paper*	N/A
HeLa GIV F1685A	*This paper*	N/A
HeLa GIV F1719A	*This paper*	N/A
HEK293T	ATCC	ATCC® CRL-11268
COS7	ATCC	ATCC® CRL-1651
DLD1	ATCC	ATCC® HTB-126
DLD1 parental and GIV KO (CRISPR Cas9) lines	Ear et al.^37^	Published in 37
MDA-MB-231	ATCC	ATCC® HTB-26
MDA-MB-231 parental and GIV KO (CRISPR Cas9) lines	*This paper*	N/A

**Recombinant DNA**		

Girdin CRISPR/Cas9 KO Plasmid (h2)	*Santa Cruz Biotechnology (SCBT) Inc*.	Sc-402236-KO-2
GST-BRCA1-CT-WT (BRCT) [pGEX-4T-human BRCA1 BRCT domain (residues 1599–1863)]	*Zhou Songyang*^76^	N/A
GST-BRCA1-CT-M1775R (BRCT) [pGEX-4T-human BRCA1 BRCT domain (residues 1599–1863)]	*This paper*	N/A
GST-BRCA1-NT(RING) [pGEX-4T-human BRCA1 RING domain (residues 1–661)]	*Zhou Songyang*^76^	N/A
GST-MDC1 [pGEX-4T-human MDC1 (residues 2727–3089)]	*Zhou Songyang*^76^	N/A
GST-DNA ligase IV [pGEX-4T-human DNA ligase IV (residues 618–911)]	*Zhou Songyang*^76^	N/A
GST-BARD1 [pGEX-4T-human BARD1 (residues 554 to 777)	*Zhou Songyang*^76^	N/A
BACH1/FANCJ myc tagged pcDNA3 vector	*David Livingston’s group*^77^	N/A
GFP-tagged CtIP	*David Livingston’s group*^78^	N/A
HA-tagged BRCA1-WT	*David Livingston’s group*^79^	N/A
pcDNA3(*BssH*II)-HA-3XFLAG-BRCA1 WT	*Kristoffer Valerie’s group*^44^	N/A
pcDNA3(*BssH*II)-HA-3XFLAG-BRCA1 K1702M	*Kristoffer Valerie’s group* Dever, 2011 #7}	N/A
CMV14-p3X FLAG-GIV WT (full length)	Garcia-Marcos et al.^20^	N/A
CMV14-p3X FLAG-GIV-F1685A (full length)	Garcia-Marcos et al.^20^	N/A
CMV14-p3X FLAG-GIV-F1719A (full length)	*This paper*	N/A
CMV14-p3X FLAG-GIV-S1716D (full length)	*This paper*	N/A
CMV14-p3X FLAG-GIV-S1716D/F1719A (full length)	*This paper*	N/A
pET-28b-GIV-CT-WT (aa 1623–1870)	Garcia-Marcos et al.^20^	N/A
pET-28b-GIV-CT-WT (aa 1660–1870)	Garcia-Marcos et al.^20^	N/A
pET-28b-GIV-CT-WT (aa 1790–1870)	Garcia-Marcos et al.^20^	N/A
pGEX-4T-GIV-CT-WT (a.a. 1623–1870)	Garcia-Marcos et al.^20^	N/A
pGEX-4T-GIV-CT-F1685A (a.a. 1623–1870)	Garcia-Marcos et al.^20^	N/A
pGEX-4T-GIV-CT-S1716A (a.a. 1623–1870)	*This paper*	N/A
pGEX-4T-GIV-CT-S1716D (a.a. 1623–1870)	*This paper*	N/A
pET-28b-GIV-CT-F1685A (aa 1660–1870)	Garcia-Marcos et al.^20^	N/A
pET-28b-GIV-CT-S1716D (aa 1660–1870)	*This paper*	N/A
pET-28b-GIV-CT-S1716A (aa 1660–1870)	*This paper*	N/A
Lentiviral vector expressing a truncated version of p53BP1 fused to mApple	*Addgene*	*69531*

**Oligos and primers**		

POLB	AGTGGGCTGGATGTAACCTG	SA-PCR (0.192 kb)
POLB	CCAGTAGATGTGCTGCCAGA	SA-PCR (0.192 kb)
HPRT	TGGGATTACACGTGTGAACCAACC	LA-qPCR (10.4 kb)
HPRT	GCTCTACCCTCTCCTCTACCGTCC	LA-qPCR (10.4 kb)
HPRT	TGCTCGAGATGTGATGAAGG	SA-PCR (0.286 kb)
HPRT	CTGCATTGTTTTGCCAGTGT	SA-PCR (0.286 kb)
POLB	CATGTCACCACTGGACTCTGCAC	LA-qPCR (12.2 kb)
POLB	CCTGGAGTAGGAACAAAAATTGCT	LA-qPCR (12.2 kb)

**Software and algorithms**		

ImageJ	National Institute of Health	https://imagej.net/Welcome
DAVID 6.8	DAVID Bioinformatics Resources	https://david.ncifcrf.gov/home.jsp
FlowJo	FlowJo, LLC	https://www.flowjo.com
Prism	GraphPad	https://www.graphpad.com/scientific-software/prism/
LAS-X	Leica	www.leica-microsystems.com/products/microscope-software/p/leica-las-x-ls
Molsoft	Molsoft, LLC	https://www.molsoft.com/index.html
Pymol	Pymol.org	https://pymol.org/2/
Illustrator	Adobe	https://www.adobe.com/products/illustrator.html
ImageStudio Lite	LI-COR	https://www.licor.com/bio/image-studio-lite/
MATLAB	MathWorks	https://www.mathworks.com/products/matlab.html

**Other**		

Prolong Glass	Thermo Fisher Scientific	P36980

### Resource availability

#### Lead contact

Further information and requests for resources and reagents should be directed to and will be fulfilled by the lead contact, Pradipta Ghosh, prghosh@ucsd.edu.

#### Materials availability

This study has generated constructs and cell lines. These materials can only be accessed through proper material transfer agreement following the guidelines of the University of California, San Diego.

### Experimental model and subject details

Human (HeLa, Cos7, HEK, Hs578T, DLD-1, and MDA-MB-231) cell lines were used in various assays. This study does not involve the use of human subjects or animals.

### Method details

#### Cell lines and culture methods

HeLa, Cos7, HEK, Hs578T, DLD-1, and MDA-MB-231 cells were grown at 37°C in their suitable media, according to their supplier instructions, supplemented with 10% FBS, 100 U/mL penicillin, 100 μg/mL streptomycin, 1% L-glutamine, and 5% CO2.

#### Plasmid constructs and mutagenesis

For mammalian expression, a well-characterized and extensively validated C-terminal FLAG-tagged construct was used.^41^^,^^67^ It was originally generated by cloning human GIV (NCBI RefSeq Accession: Q3V6T2) into p3XFLAG-CMV-14 between NotI and BamHI. All subsequent site-directed mutagenesis (GIV-Flag full-length F1685A, F1719A, S1716A (SA), and S1716D (SD) were carried out on this template using Quick Change as per the manufacturer’s protocol. For BRCA1 constructs, the sources are listed in the “Table of key resources table”. The target mutants for GIV and BRCA1 were confirmed by sequencing. The GST-BRCA1-WT, GST-BRCA1-CT (BRCT), GST-BRCA1-NT, GST-BRCA1-K1702M, GST-BRCA1-M1775R, GST-MDC1, GST-DNA ligase IV, GST-BARD1, GST-GIV CT, GST-GIV WT, GST-GIV F1685A, GST-GIV F1719A, GST-GIV-CT- S1716A, GST-GIV-CT- S1716D, His GIV-CT, and GST-Gαi3 fusion proteins were used for *in vitro* protein-protein interaction.

#### Transfection, generation of stable cell lines and cell lysis

Transfection was carried out using Genejuice (Novagen) for DNA plasmids following the manufacturers’ protocols. Hela cell lines stably expressing GIV constructs (WT, F1685A, and F1719A) were selected after transfection in the presence of 800 mg/mL G418 for 6 weeks. The resultant multiclonal pool was subsequently maintained in the presence of 500 mg/mL G418. GIV expression was verified independently by immunoblotting using anti-GIV antibody. Whole-cell lysates were prepared after washing cells with cold PBS prior to resuspending and boiling them in sample buffer. Lysates used as a source of proteins in immunoprecipitation or pulldown assays were prepared by resuspending cells in Tx-100 lysis buffer [20 mM HEPES, pH 7.2, 5 mM Mg-acetate, 125 mM K-acetate, 0.4% Triton X-100, 1 mM DTT, supplemented with sodium orthovanadate (500 mM), phosphatase (Sigma) and protease (Roche) inhibitor cocktails], after which they were passed through a 28G needle at 4°C, and cleared (10,000 × g for 10 min) before use in subsequent experiments.

#### Biotin proximity labeling

BioID was performed as previously described.^80^ Briefly, HEK293T were plated 24 hrs prior to transfection with mycBirA-tagged GIV construct. Thirty hours post transfection, cells were treated with 50 μM biotin (dissolved in culture media) for 16 hrs. Cells were then rinsed two times with PBS and lysed by resuspending in lysis buffer (50 mM Tris, pH 7.4, 500 mM NaCl, 0.4% SDS, 1 mM dithiothreitol, 2% Triton X-100, and 1× Complete protease inhibitor) and sonication in a bath sonicator. Cell lysates were then cleared by centrifugation at 20,000 X g for 20 mins and supernatant was then collected and incubated with streptavidin magnetic beads overnight at 4°C. After incubation, beads were washed twice with 2% SDS, once with wash buffer 1 (0.1% deoxycholate, 1% Triton X-100, 500 mM NaCl, 1 mM EDTA, and 50 mM HEPES, pH 7.5), followed with once wash using wash buffer 2 (250 mM LiCl, 0.5% NP-40, 0.5% deoxycholate, 1 mM EDTA, and 10 mM Tris, pH 8.0), and once with 50 mM Tris pH 8.0. Biotinylated complexes were then eluted using sample buffer containing excess biotin and heating at 100°C. Prior to mass spectrometry identification, eluted samples were run on SDS-PAGE and proteins were extracted by in gel digest.

#### In gel digest

Protein digest and mass spectrometry was perform as previously described.^81^ Briefly, the gel slices were cut into 1 mm × 1 mm cubes, destained 3 times by first washing with 100 μl of 100 mM ammonium bicarbonate for 15 minutes, followed by the addition of equal volume acetonitrile (ACN) for 15 minutes. The supernatant was collected, and samples were dried using a speedvac. Samples were then reduced by mixing with 200 μL of 100 mM ammonium bicarbonate-10 mM DTT and incubated at 56°C for 30 minutes. The liquid was removed and 200 μl of 100 mM ammonium bicarbonate-55mM iodoacetamide was added to gel pieces and incubated covered at room temperature for 20 minutes. After the removal of the supernatant and one wash with 100 mM ammonium bicarbonate for 15 minutes, equal volume of ACN was added to dehydrate the gel pieces. The solution was then removed, and samples were dried in a SpeedVac. For digestion, enough solution of ice-cold trypsin (0.01 μg/μl) in 50 mM ammonium bicarbonate was added to cover the gel pieces and set on ice for 30 min. After complete rehydration, the excess trypsin solution was removed, replaced with fresh 50 mM ammonium bicarbonate, and left overnight at 37°C. The peptides were extracted twice by the addition of 50 μL of 0.2% formic acid and 5% ACN and vortex mixing at room temperature for 30 min. The supernatant was removed and saved. A total of 50 μL of 50% ACN-0.2% formic acid was added to the sample, and vortexed again at room temperature for 30 min. The supernatant was removed and combined with the supernatant from the first extraction. The combined extractions are analyzed directly by liquid chromatography (LC) in combination with tandem mass spectroscopy (MS/MS) using electrospray ionization.

#### LC-MS analysis

Trypsin-digested peptides were analyzed by ultra-high-pressure liquid chromatography (UPLC) coupled with tandem mass spectroscopy (LC-MS/MS) using nano-spray ionization. The nanospray ionization experiments were performed using a Orbitrap fusion Lumos hybrid mass spectrometer (Thermo) interfaced with nano-scale reversed-phase UPLC (Thermo Dionex UltiMate™ 3000 RSLC nano System) using a 25 cm, 75-micron ID glass capillary packed with 1.7-μm C18 (130) BEH^TM^ beads (Waters corporation). Peptides were eluted from the C18 column into the mass spectrometer using a linear gradient (5–80%) of ACN (Acetonitrile) at a flow rate of 375 μL/min for 1h. The buffers used to create the ACN gradient were: Buffer A (98% H_2_O, 2% ACN, 0.1% formic acid) and Buffer B (100% ACN, 0.1% formic acid). Mass spectrometer parameters are as follows; an MS1 survey scan using the orbitrap detector (mass range (m/z): 400–1500 (using quadrupole isolation), 120000 resolution setting, spray voltage of 2200 V, Ion transfer tube temperature of 275 C, AGC target of 400000, and maximum injection time of 50 ms) was followed by data dependent scans (top speed for most intense ions, with charge state set to only include +2–5 ions, and 5 second exclusion time, while selecting ions with minimal intensities of 50000 at in which the collision event was carried out in the high energy collision cell (HCD Collision Energy of 30%), and the fragment masses where analyzed in the ion trap mass analyzer (With ion trap scan rate of turbo, first mass m/z was 100, AGC Target 5000 and maximum injection time of 35ms). Protein identification and label free quantification was carried out using Peaks Studio 8.5 (Bioinformatics solutions Inc.). The proteomic dataset was deposited to the ProteomeXchange Consortium *via* the PRIDE partner repository with the data set identifier PXD022601.^37^

#### Gene ontology analysis

Identified proteins by mass spec. anaylsis unique to plus biotin samples, but not in minus biotin samples, were analyzed using DAVID and functional annotation was grouped by molecular function and cellular component for GO analysis. Classification with p-value less than 0.05 were considered as significant.

#### GIV CRISPR/Cas9 gene editing and validation

Pooled guide RNA plasmids (commercially obtained from Santa Cruz Biotechnology; Cat# sc-402236-KO-2) were used. These CRISPR/Cas9 KO plasmids consists of GFP and girdin-specific 20 nt guide RNA sequences derived from the GeCKO (v2) library and target human Girdin exons 6 and 7. Plasmids were transfected into Hela and MDA-MB-231 cells using PEI. Cells were sorted into individual wells using a cell sorter based on GFP expression. To identify cell clones harboring mutations in gene coding sequence, genomic DNA was extracted using 50 mM NaOH and boiling at 95°C for 60 mins. After extraction, pH was neutralized by the addition of 10% volume 1.0 M Tris-pH 8.0. The crude genomic extract was then used in PCR reactions with primers flanking the targeted site. Amplicons were analyzed for insertions/deletions (indels) using a TBE-PAGE gel. Indel sequence was determined by cloning amplicons into a TOPO-TA cloning vector (Invitrogen) following manufacturer’s protocol. DLD1 parental and GIV KO lines were generated and validated as described before.^37^

#### Protein expression and purification

GST and His-tagged recombinant proteins were expressed in *E*. *coli* strain BL21 (DE3) (Invitrogen) and purified as described previously.^20^^,^^21^^,^^67^ Briefly, bacterial cultures were induced overnight at 25°C with 1 mM isopropylb-D-1-thio-galactopyranoside (IPTG). Pelleted bacteria from 1 L of culture were resuspended in 20 mL GST-lysis buffer [25 mM TrisHCl, pH 7.5, 20 mM NaCl, 1 mM EDTA, 20% (vol/vol) glycerol, 1% (vol/vol) Triton X-100, 2X protease inhibitor mixture (Complete EDTA-free; Roche Diagnostics)] or in 20 mL His-lysis buffer [50 mM NaH2PO4 (pH 7.4), 300 mM NaCl, 10 mM imidazole, 1% (vol/vol) Triton X-100, 2X protease inhibitor mixture (Complete EDTA-free; Roche Diagnostics)] for GST or His-fused proteins, respectively. After sonication (three cycles, with pulses lasting 30 s/cycle, and with 2 min intervals between cycles to prevent heating), lysates were centrifuged at 12,000X g at 4°C for 20 min. Solubilized proteins were affinity purified on glutathione-Sepharose 4B beads (GE Healthcare) or HisPur Cobalt Resin (Pierce), dialyzed overnight against PBS, and stored at 80°C.

#### In vitro GST-Pulldown and in-cellulo Co-immunoprecipitation (CoIP) assays

Purified GST-tagged proteins from *E*. *coli* were immobilized onto glutathione-Sepharose beads and incubated with binding buffer (50 mM Tris-HCl (pH 7.4), 100 mM NaCl, 0.4% (v:v) Nonidet P-40, 10 mM MgCl2, 5 mM EDTA, 2 mM DTT) for 60 mins at room temperature. For the pulldown of protein-protein complexes from cell lysates, cells were first lysed in cell lysis buffer (20 mM HEPES, pH 7.2, 5 mM Mg-acetate, 125 mM K-acetate, 0.4% Triton X-100, 1 mM DTT, 500 μM sodium orthovanadate, phosphatase inhibitor cocktail (Sigma-Aldrich) and protease inhibitor cocktail (Roche)) using a 28G needle and syringe, followed by centrifugation at 10,000Xg for 10 mins. Cleared supernatant was then used in binding reaction with immobilized GST-proteins for 4 hours at 4°C. After binding, bound complexes were washed four times with 1 ml phosphate wash buffer (4.3 mM Na2HPO4, 1.4 mM KH2PO4, pH 7.4, 137 mM NaCl, 2.7 mM KCl, 0.1% (v:v) Tween 20, 10 mM MgCl2, 5 mM EDTA, 2 mM DTT, 0.5 mM sodium orthovanadate). Bound proteins were then eluted through boiling at 100°C in sample buffer.

For CoIP assays, cells lysates (as prepared above) was incubated with capture antibodies for 3 hours at 4°C, followed by the addition of Protein A or Protein G beads to capture antibody bound protein-protein complexes. Bound proteins were then eluted through boiling at 100°C in sample buffer.

#### Quantitative immunoblotting

For immunoblotting, protein samples were boiled in Laemmli sample buffer, separated by SDS-PAGE and transferred onto 0.4 μm PVDF membrane (Millipore) prior to blotting. Post transfer, membranes were blocked using 5% Non-fat milk or 5% BSA dissolved in PBS. Primary antibodies were prepared in blocking buffer containing 0.1% Tween-20 and incubated with blots, rocking overnight at 4°C. After incubation, blots were incubated with secondary antibodies for one hour at room temperature, washed, and imaged using a dual-color Li-Cor Odyssey imaging system.

#### Immunofluorescence and confocal microscopy, image analysis

Cells were fixed using −20°C methanol (or 4°C paraformaldehyde, PFA) for 20 to 30 min, rinse with PBS, and then permeabilized for 1h using blocking/permeabilization buffer (0.4% Triton X-100 and 2 mg/mL BSA dissolved in PBS). Primary antibody and secondary antibody were diluted in blocking buffer and incubated with cells for 1 hr each. Coverslips were mounted using Prolong Gold (Invitrogen) and imaged using a Leica SPE CTR4000 confocal microscope.

#### MTT assay

Cell proliferation was measured using the MTT reagent and cells cultured in 96-well plates. Parental or GIV-KO HeLa, DLD1 or MDA-MB-231 cells or HeLa GIV-KO cells stably expressing WT GIV, GIV F1685A, or GIV F1719A were cultured and treated with different concentrations of Doxorubicin (0.1, 0.25, 0.5, 1, 2, and 4 μM), cisplatin (1, 5, 10, 25, 50, and 100 μM), or etoposide (1, 5, 10, 25, 50, and 100 μM) using DMSO as a negative control for 36 h. MDA-MB-231 and DLD-1 cell lines were treated with only dox (0.5 μM) for 24 h. Then the cell lines were incubated with MTT for 4 hr at 37°C. After incubation, culture media was removed, replaced with phosphate buffered saline (PBS) and 150 μL of DMSO was added in order to solubilize the MTT formazan crystals. Optical density was determined at 590 nm using a TECAN plate reader. At least three independent experiments were performed.

#### Anchorage-dependent colony formation assay

Anchorage-dependent growth was monitored as described previously.^21^^,^^82^ Briefly, anchorage-dependent growth was monitored on solid (plastic) surface. Approximately 2,000 parental or GIV-KO HeLa cells or GIV-KO cells stably expressing WT GIV, GIV F1685A, or GIV F1719A were plated in 6-well plates and incubated in 5% CO2 at 37°C for ∼2 weeks in 2% FBS growth media in the presence of 10 nM Dox. After every three days, media were changed with fresh media containing 10 nM Dox. Colonies were then stained with 0.005% crystal violet for 1 hr. Entire plate surface area was scored for colonies and each treatment was done in triplicate and repeated thrice.

#### Cell cycle and apoptosis analyses

Cell cycle analysis and apoptotic cell quantification was performed using the Guava cell cycle reagent (Millipore Sigma) or the annexin V/propidium iodide (PI) staining kit (Thermo Fisher Scientific), respectively, according to the manufacturer’s instructions. Cells were quantified on a BD LSR II flow cytometer and analyzed using FlowJo software (FlowJo, Ashland, OR, USA).

#### Long amplicon PCR

Genomic DNA extraction was performed using the genomic-tip 20/G kit (Qiagen, Cat no. 10223, with corresponding buffer sets) per the manufacturer’s directions. This kit has the advantage of minimizing DNA oxidation during the isolation steps, and thus it can be used reliably for isolation of high molecular weight DNA with excellent template integrity to detect endogenous DNA damage using LA-qPCR. After precise quantitation of the DNA by Pico Green (Invitrogen Cat no. P7589) in a 96-well black-bottomed plate, the genomic DNA (500 ng) was digested with the *E*. *coli* enzymes Fpg and Nei (New England Biolabs) in reaction volume of 50 μL using Buffer 1 from NEB (with 1 mM MgCl2) as the common buffer to induce strand breaks at the sites of the unrepaired oxidized base lesion. Gene-specific LA-qPCR analyses for measuring DNA damage were performed using Long Amp Taq DNA polymerase (New England Biolabs, Cat no MO323S). The numbers of cycles and DNA concentrations were standardized in each case before the actual reaction, so that the PCR remains within the linear range of amplification. The final PCR condition was optimized at 94 °C for 30 s (94 °C for 30 s, 55–60 °C for 30 s depending on the oligo annealing temperature, 65 °C for 10 min) for 25 cycles and 65 °C for 10 min. 25 ng of DNA template was used in each case, and the LA-qPCR was set for all the genes under study from the same stock of Fpg/Nei-treated diluted genomic DNA samples to avoid variations in PCR amplification due to sample preparation. Since amplification of a small region would be independent of DNA damage, a small DNA fragment for each gene was also amplified to normalize the amplification of large fragments. The PCR conditions were 94 °C for 30 s (94 °C for 30 s, 54–58°C for 20 s, and 68 °C for 30 s) for 25 cycles and 68 °C for 5 min. 25 ng of template from the same Fpg/Nei digested DNA aliquot was used for short PCR using green mix (NEB). The amplified products were then visualized on gels and quantitated with an ImageJ automated digitizing system (National Institutes of Health) based on three independent replicate PCRs. The extent of damage was calculated.

#### Stable cell lines with p53BP1 fluorescent reporter

We obtained a lentiviral vector expressing a truncated version of p53BP1 fused to mApple from Dr. Ralph Weissleder, Massachusetts General Hospital, USA (Addgene #69531).^54^ To establish MDA-MB-231 and HeLa parental and GIV knockout cells stably expressing the reporter, we produced recombinant lentiviruses and transduced each cell line as described previously.^83^ We selected batch populations of transduced cells with 5 μg/mL puromycin. After selection, we maintained cells in DMEM (#10313, Gibco, Thermo Fisher, Grand Island, NY, USA) with 10% FBS (HyClone, ThermoScientific, Waltham, MA, USA), 1% GlutaMAX (#35050, Gibco), and 1% Penicillin-Streptomycin (P/S, #15140, Gibco) and no added puromycin.

#### Image acquisition and analysis

We seeded cells in 6- or 96-well glass bottom plates (P-96-1.5H-N or P06-1.5H-N, Cellvis, Mountain View, CA, USA) at densities of 1.25 × 105 or 5 × 103 cells/well, respectively, in imaging base medium (FluoroBrite DMEM media (A1896701, ThermoFisher Scientific, Waltham, MA USA), 1% GlutaMax, 1% PenStrep, 1% sodium pyruvate, and 10% FBS (HyClone)). One day later, we acquired baseline pre-treatment images and then changed to fresh imaging base medium containing types and concentrations of chemotherapy drugs indicated in figure legends. We repeated imaging after one day of treatment and in selected experiments imaged again after two days. We performed imaging studies with an EVOS M7000 Imaging System (ThermoFisher), 40X objective, and the RFP cube for the instrument. During imaging, we used incubator conditions of 37 ͦ C, 5% CO2, and 80% humidity. We randomly acquired Z-stack images (7–9 planes at ∼ 0.8 μm intervals) from 6-8 fields per condition, which typically encompassed > 100 cells each. To accurately quantify the number of bright P53BP1 foci per cell nucleus, we developed custom MATLAB image processing and analysis software. To process the raw images, we first smoothed each plane with an edge-preserving Gaussian bilateral filter and then calculated a maximum intensity projection (MIP) from these images. We created a binary mask of nuclei from the MIP by normalizing the MIP intensities from 0 to 1 and applying Otsu’s method of binarization with adaptive thresholding. We then filtered this mask for objects of the appropriate area and filled any voids in the mask. To identify the bright foci within nuclei, we created a separate mask from the normalized MIP using an extended maxima transform followed by a filter for objects in the binary mask of the appropriate area. Our software automatically tabulated the number of bright foci within each nucleus for each cell in the image and aggregated data for images within each well.

#### G protein activation assay

For immunoprecipitation of active Gαi3, freshly prepared cell lysates (2–4 mg) were incubated for 30 min at 4°C with the conformational Gαi3:GTP mouse antibody (1 μg)^84^ or pre-immune control mouse IgG. Protein G Sepharose beads (GE Healthcare) were added and incubated at 4°C for additional 30 min (total duration of assay is 1 h). Beads were immediately washed 3 times using 1 mL of lysis buffer (composition exactly as above; no nucleotides added) and immune complexes were eluted by boiling in SDS as previously described. Previous work from our lab validated the use of this antibody to selectively immunoprecipitate His-Gαi3 recombinant proteins loaded with GTP (physiologic active conformation that is transient) and GTPϒS (non-hydrolysable nucleotide mimicking a stable active conformation) but not GDP (inactive conformation).^85^

#### Homology modeling

The prediction the protein and GIV peptide docked interface was performed by using CABSDOCK^86^ web server and the final representation was using Pymol visualization tool.^87^ For the analysis the PDB structures 1T19, 1t2V, 1N5O were taken into consideration. 10 residues of GIV “SLSVSSDFLGKD” for their secondary structure prediction was tested on PSIPRED.^88^ The visualization of PDB:1T29 and PDB:1N5O structures was done using MolSoft LLC (https://www.molsoft.com/).

#### Image processing

All images were processed on ImageJ software (NIH) or FLOWJO software and assembled into figure panels using Photoshop and Illustrator (Adobe Creative Cloud). All graphs were generated using GraphPad Prism.

### Quantification and statistical analysis

#### Statistical analysis and replicates

All experiments were repeated at least three times, and results were presented either as average ±SEM. Statistical significance was assessed using one-way analysis of variance (ANOVA) including a Tukey’s test for multiple comparisons. ∗p < 0.05, ∗∗p < 0.01, ∗∗∗p < 0.001, ∗∗∗∗p < 0.0001.

## Data Availability

• All data is available in the main text or the supplemental information. Original western blot images and microscopy data will be shared by the lead contact upon request. This paper also analyzes an existing, publicly available proteomics dataset. Accession number for this dataset is listed in the key resources table. Source data for gene ontology analyses are provided with this paper. • This paper does not report original code. • Any additional information required to reanalyze the data reported in this paper is available from the lead contact upon request.
