# Supplementary material for: Regulation of DNA damage response by trimeric G-proteins
Source: iScience. 2023 Jan 13;26(2):105973. doi: 10.1016/j.isci.2023.105973 (PMC9900518; doi:10.1016/j.isci.2023.105973)
Supplement: Document S1. Figures S1–S7 and Tables S1–S4 [file mmc1.pdf]

iScience, Volume *26*

Supplemental information

Regulation of DNA damage response by trimeric G-proteins

Amer Ali Abd El-Hafeez, Nina Sun, Anirban Chakraborty, Jason Ear, Suchismita Roy, Pranavi Chamarthi, Navin Rajapakse, Soumita Das, Kathryn E. Luker, Tapas K. Hazra, Gary D. Luker, and Pradipta Ghosh

Regulation of DNA damage response by trimeric G-proteins

Amer Ali Abd El-Hafeez[1, ¶], Nina Sun[1], Anirban Chakraborty[2], Jason Ear[1,3], Suchismita Roy[1], Pranavi Chamarthi[1], Navin Rajapakse[1], Soumita Das[4], Kathryn E Luker[5], Tapas K. Hazra[2], Gary D. Luker[5-7], Pradipta Ghosh[1, 8-11§]

[1]Department of Cellular and Molecular Medicine, University of California San Diego, La Jolla, California 92093, USA

[2]Department of Internal Medicine, University of Texas Medical Branch, Galveston, Texas 77555, USA

[3]Biological Sciences Department, California State Polytechnic University, Pomona, California 91768, USA

[4]Department of Pathology, University of California San Diego, La Jolla, California 92093, USA

[5]Center for Molecular Imaging, Department of Radiology, University of Michigan 109 Zina Pitcher Place, Ann Arbor, MI, 48109-2200, USA

[6]Department of Biomedical Engineering, University of Michigan, 2200 Bonisteel, Blvd., Ann Arbor, MI, 48109-2099, USA.

[7]Department of Microbiology and Immunology, University of Michigan, 109 Zina Pitcher Place, Ann Arbor, MI, 48109-2200, USA

[8]Department of Medicine, University of California San Diego, La Jolla, California 92093, USA

[9]Moores Comprehensive Cancer Center, University of California San

Diego, La Jolla, California 92093, USA

[10]Veterans Affairs Medical Center, La Jolla, CA, USA.

[11]Lead Contact

[§]Corresponding author. prghosh@ucsd.edu

¶ **Secondary Affiliation**: Pharmacology and Experimental Oncology Unit, Cancer Biology Department, National Cancer Institute, Cairo University, Cairo, Egypt

INVENTORY OF SUPPLEMENTARY MATERIALS

- **SUPPLEMENTARY FIGURES AND LEGENDS (7)**
- **SUPPLEMENTARY TABLES (4)**

SUPPLEMENTARY FIGURES AND LEGENDS

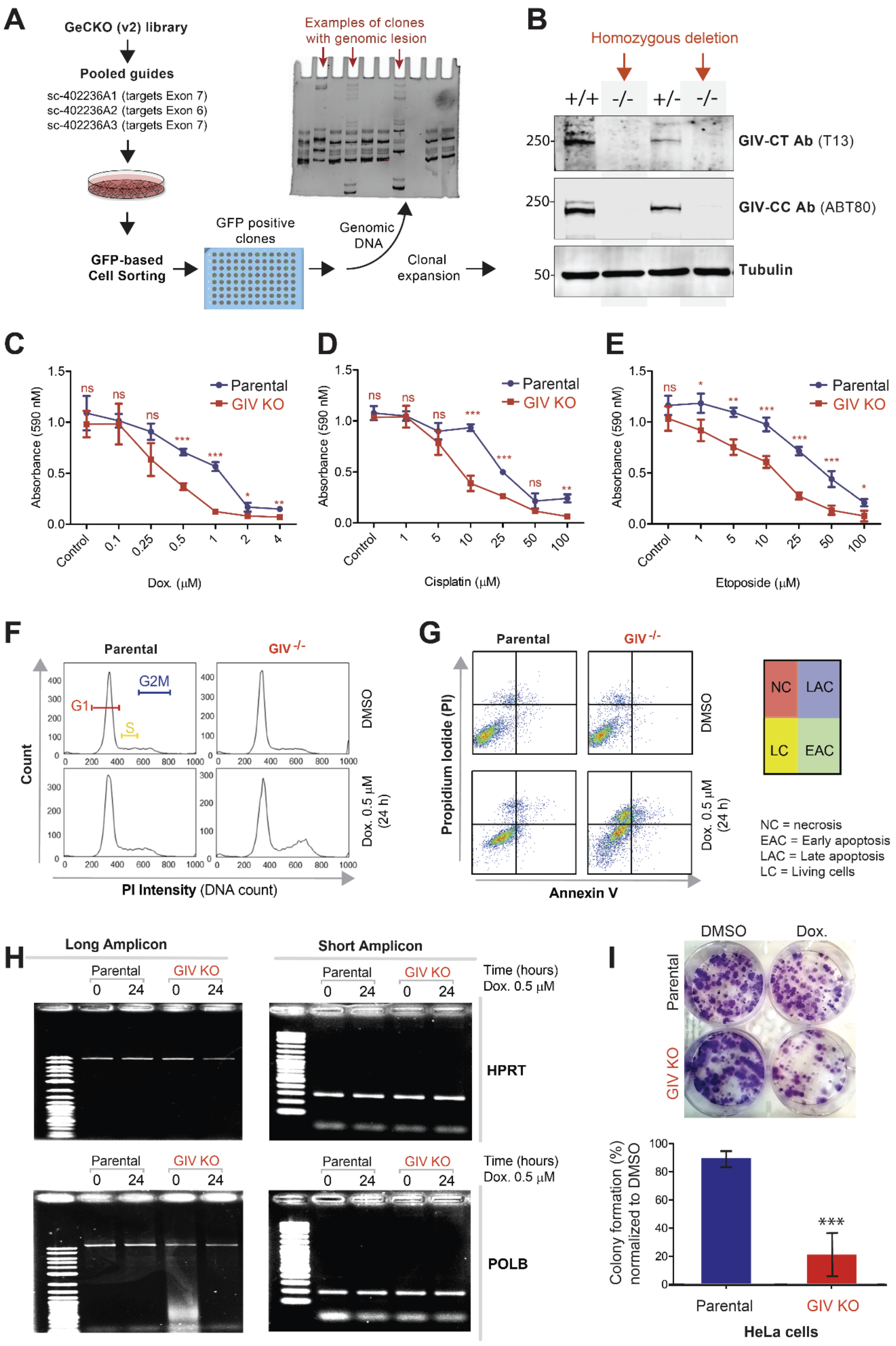

Figure S1 [related to Figure 2]. DNA damage repair response is impaired in cells without GIV.

A-B. Steps leading to creation of GIV KO HeLa lines. Cas9 target guides against GIV (CCDC88A) exons are listed in A. Gel showing screening of colonies by PCR of region flanking target site using genomic DNA from various cell clones from Cas9 selection (*bottom*). Immunoblots in B show examples of colonies that were either homozygous (-/-) or heterozygous (-/+) KO for GIV allele. KO clones were pooled to avoid clonal bias.
C-E. Line graphs display the metabolically active parental (blue) and GIV KO (red) cells that survived various doses of Doxorubicin (C), Cisplatin (D) or Etoposide (E), as determined by *MTT* tetrazolium assay (see *Methods*). Data displayed as mean ± S.E.M. and t-test was used to determine significance. (*; $p \leq 0.05$; **$p < 0.01$, ***$p < 0.001$. ns = not significant). See also Fig 2C for the table of IC50 values.
F. Histograms show the percentage of cells at various stages of cell cycle (G1, S and G2/M) after challenged with Dox or vehicle control (DMSO). See bar graphs in Fig 2D for quantification.
G. Necrosis (NC), apoptotic (early, EAC; late, LAC; or combined) or living cells (LC) were quantified after challenged with either Dox or vehicle control (DMSO), as assessed by annexin V staining and flow cytometry. Scatter plots are displayed. Color coded quadrants are labeled.
H. Long amplicon qPCR (LA-QPCR) was used to evaluate genomic DNA SB levels in control vs. GIV KO cells. Representative full-length gels showing PCR-amplified fragments of the *HPRT* (E, top panel) and *POLB* (E, bottom panel) genes.
I. Images (top) and bar graphs (bottom) display anchorage-dependent growth into colonies after ~2 weeks of prolonged exposure of HeLa parental and GIV KO cells to 10 nM Doxorubicin or vehicle (DMSO) control (see *Methods*).

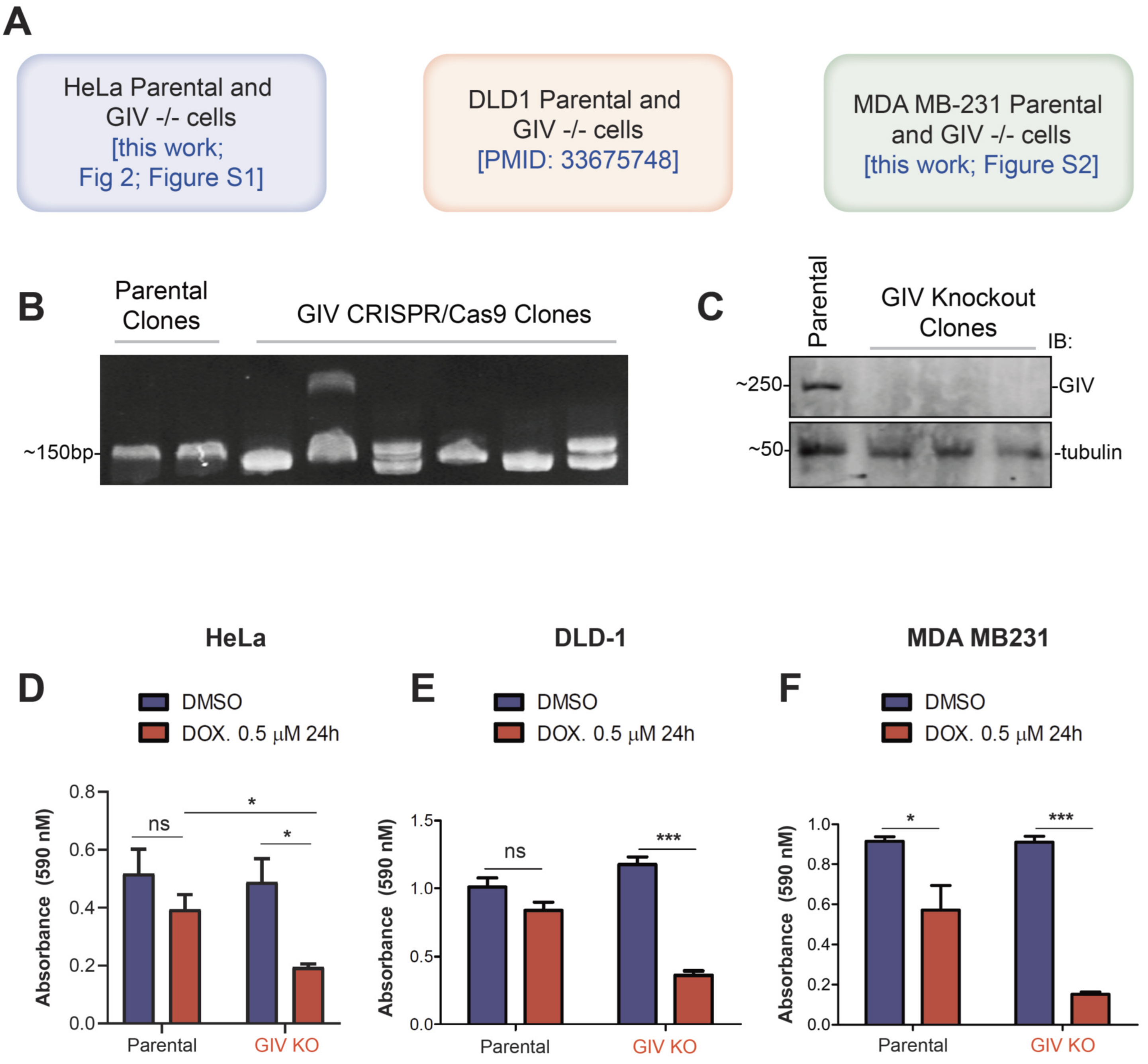

Figure S2 [related to Figure 2]. Survival after DNA damage is impaired in DLD1 and MDA-MB-231 cells without GIV.
A. Schematic outlining the three cell lines used in this work to study the role of GIV in cell survival after DNA damage. Parental and GIV KO HeLa cells are developed and validated in this work (see **Figure S1A-B**). Parental and GIV KO DLD1 cells were developed and validated in a prior work (see Key Resource Table). Generation and validation of parental and GIV KO MDA-MB-231 cells is described in **B-C. B-C.** Steps leading to the creation of GIV KO MDA-MB-231 lines. Cas9 target guides against GIV (CCDC88A) exons listed in **Fig S1A** (see *Methods*) were used to create GIV KO lines. Gel (B) showing screening of colonies by PCR of region flanking target site using genomic DNA from various cell clones from Cas9 selection. Immunoblots in C show examples of colonies that were KO for GIV allele. KO clones were pooled to reduce clonal bias. **D-F**. Bar graphs showing % survival of metabolically active HeLa (D), DLD-1 (E) and MDA-MB-231 (F) cell lines, challenged with either Dox or vehicle control (DMSO) for 24 h, as determined by *MTT* tetrazolium *assay.* Data displayed as mean ± S.E.M. and one-way ANOVA using Tukey's multiple comparisons test was used to determine significance. (*; $p \leq 0.05$; ***$p < 0.001$. ns = not significant).

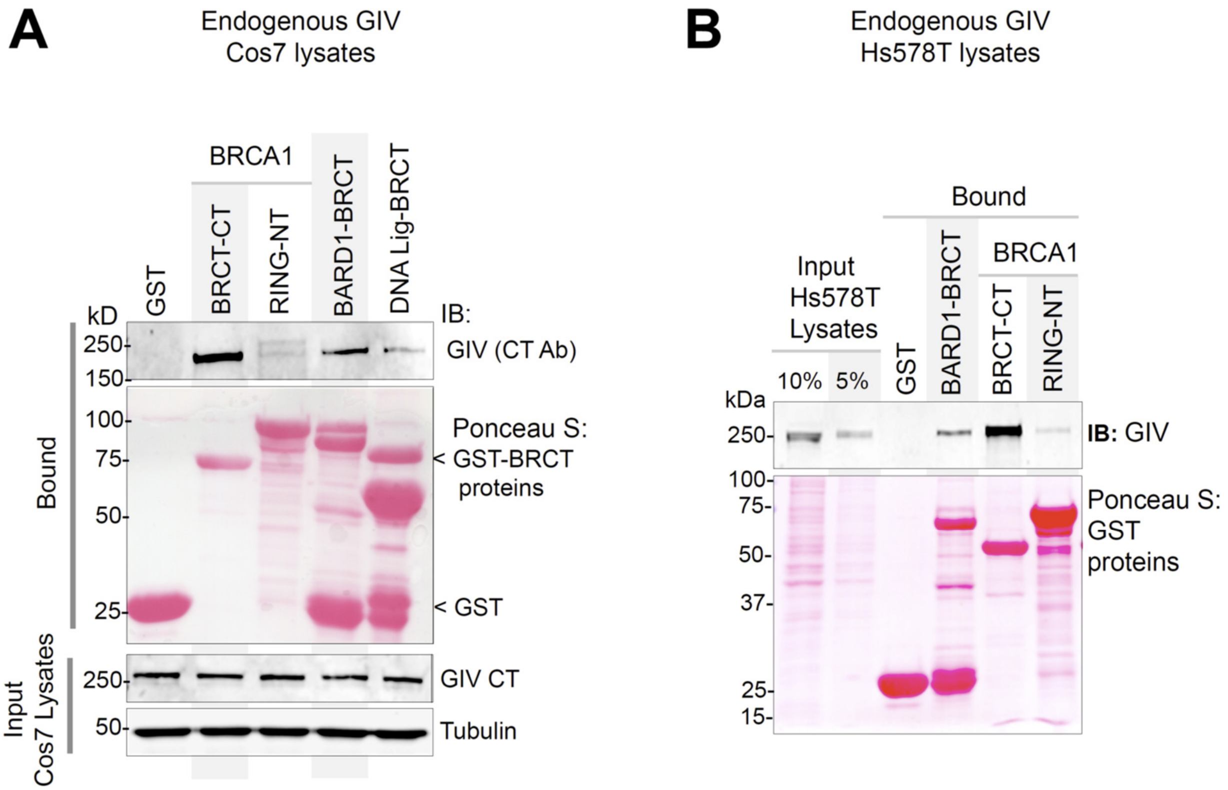

Figure S3 [related to Figure 3]. The C-terminal BRCT module of BRCA1 binds to full length GIV from lysates of Cos7 and HeLa cells. Pulldown assays were carried out using lysates of Cos7 (A) and Hs578T (B; a triple negative breast cancer line) cells as source of endogenous full length GIV with GST-BRCA1 and BARD1. Bound GIV was visualized by immunoblotting. See also **Fig 3D** for similar studes with lysates of HeLa cells.

A

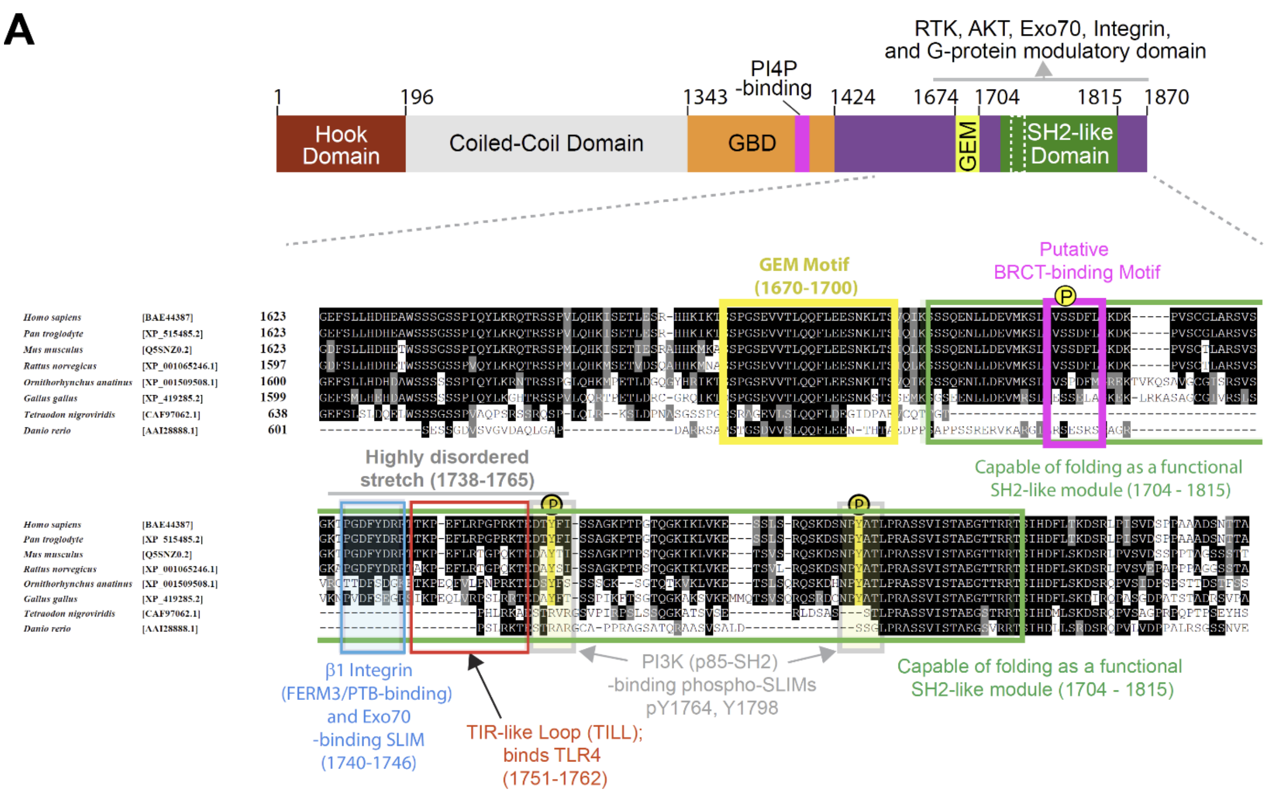

B

Girdin (human)	S171 6-p	VMKSLsVsSDFLGKD	Links	1, 2, 3, 5, 7, 8, 9
Girdin (mouse)	S171 6-p	VMKSLsVssDFLGKD	Links	3, 4, 6

1	Mertins P, et al. (2016) Proteogenomics connects somatic mutations to signalling in breast cancer. Nature 534 , 55 -62<br>27251275 Curated Info
2	Stuart SA, et al. (2015) A Phosphoproteomic Comparison of B -RAFV600E and MKK1/2 Inhibitors in Melanoma Cells. Mol Cell Proteomics 14 , 1599 -615<br>25850435 Curated Info
3	Mertins P, et al. (2014) Ischemia in tumors induces early and sustained phosphorylation changes in stress kinase pathways but does not affect global protein levels. Mol Cell Proteomics 13 , 1690 -704<br>24719451 Curated Info
4	Humphrey SJ, et al. (2013) Dynamic Adipocyte Phosphoproteome Reveals that Akt Directly Regulates mTORC2. Cell Metab 17 , 1009 -20<br>23684622 Curated Info
5	Zhou H, et al. (2013) Toward a comprehensive characterization of a human cancer cell phosphoproteome. J Proteome Res 12 , 260 -71<br>23186163 Curated Info
6	Wu X, et al. (2012) Investigation of receptor interacting protein (RIP3) -dependent protein phosphorylation by quantitative phosphoproteomics. Mol Cell Proteomics 11 , 1640 -51<br>22942356 Curated Info
7	Beli P, et al. (2012) Proteomic Investigations Reveal a Role for RNA Processing Factor THRAP3 in the DNA Damage Response. Mol Cell 46 , 212 -25<br>22424773 Curated Info
8	Possemato A (2009) CST Curation Set: 7403; Year: 2009; Biosample/Treatment: cell line, NCI - H2228 /untreated; Disease: non -small cell lung cancer; SILAC: -; Specificities of Antibodies Used to Purify Peptides prior to LCMS: (K/R)XXST<br>Curated Info
9	Brill LM, et al. (2009) Phosphoproteomic analysis of human embryonic stem cells. Cell Stem Cell 5 , 204 - 13<br>19664994 Curated Info

Figure S4 [related to Figure 3]. Discovery of an evolutionarily conserved putative BRCT-binding motif on the C terminus of GIV. A. Short linear interaction motifs (SLIMs) within GIV's C-terminus. **Top:** Bar diagram showing the various domains of GIV. GBD, G protein binding domain; GEM, Guanine nucleotide exchange modulator; SH2, Src-like homology; PI4P, phosphoinositol-4-phosphate. **Bottom**: Sequence of GIV's C-terminus showing all currently identified SLIMs. The putative BRCT-binding SLIM is highlighted in pink. **B**. A curated list of studies that reported phosphorylation at Ser1716 on GIV (Girdin). Source: Phosphosite.org, a database that was developed with grants from the NIH.

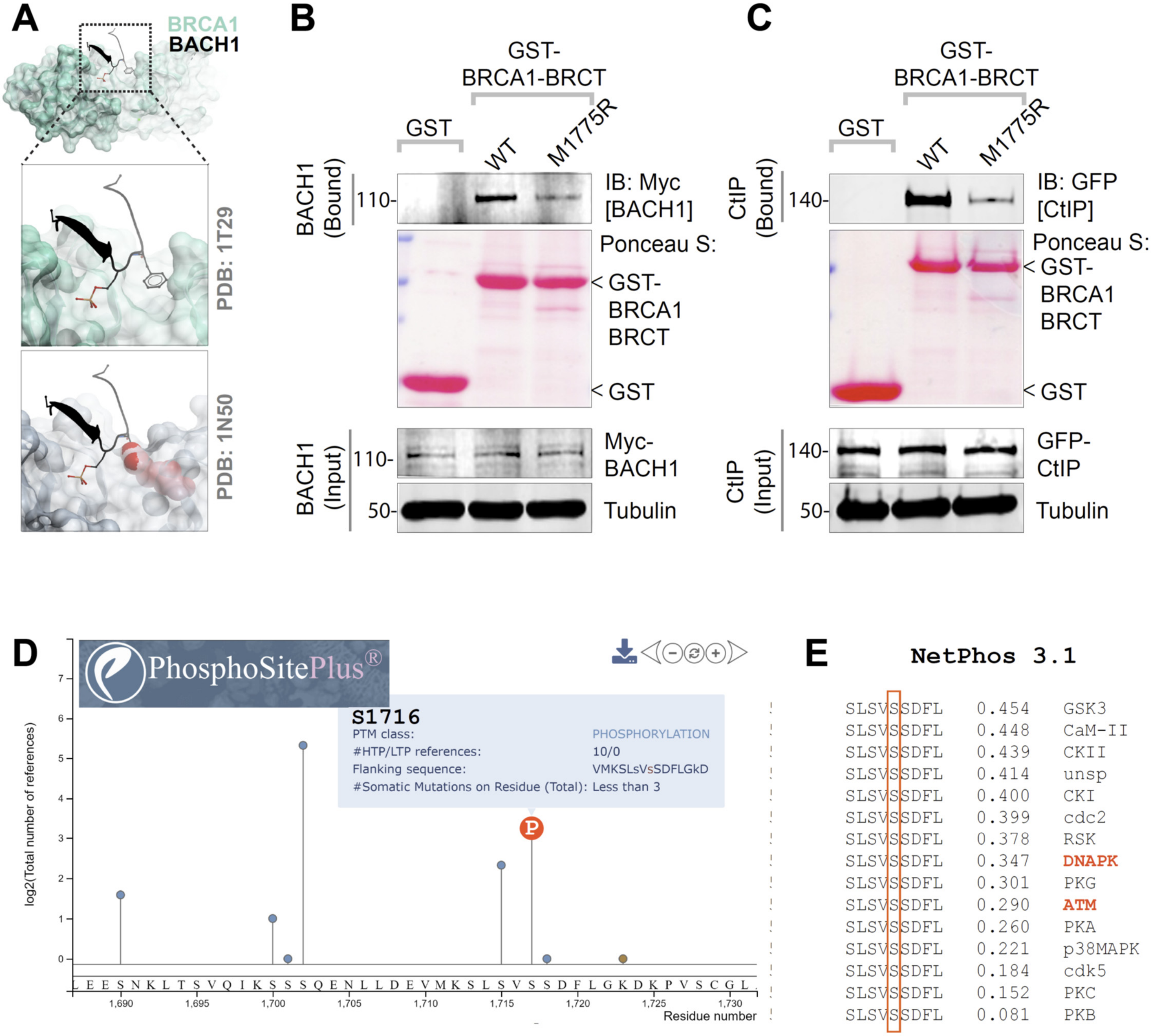

Figure S5 [related to Figure 4]. Canonical (phosphodependent) binding of CtIP and BACH1 proteins to BRCA1 and proposed mechanism of phospho-dependent binding of GIV. A. Top: Solved structure of BACH1-derived SxxF consensus-bearing phosphopeptide bound to BRCA1 BRCT modules. Bottom: Model of M1775R mutation in BRCA (from solved structure of the mutant BRCA1; PDB: 1N50) showing steric clash of Arg at 1775 precluding binding of Phe (F) within the SxxF motif. **B**. Pulldown assays were carried out using lysates of HEK cells as source of myc-BACH1 (B) or GFP-CtIP (C) and recombinant GST/GST-BRCA1 WT and M1775R mutant proteins. Bound proteins were visualized by immunoblotting with anti-myc (BACH1; B), or anti-GFP (CtIP; C) IgGs. **D**. A lollipop graph showing the number of independent mass spectrometry studies that reported phosphorylation at Ser1716 on GIV (Girdin). Source: Phosphosite.org, a database that was Developed with grants from the NIH. **E**. Predicted kinases that mediate such phosphorylation, as determined using the bioinformatic web-based resource, NetPhos 3.1 (https://services.healthtech.dtu.dk/).

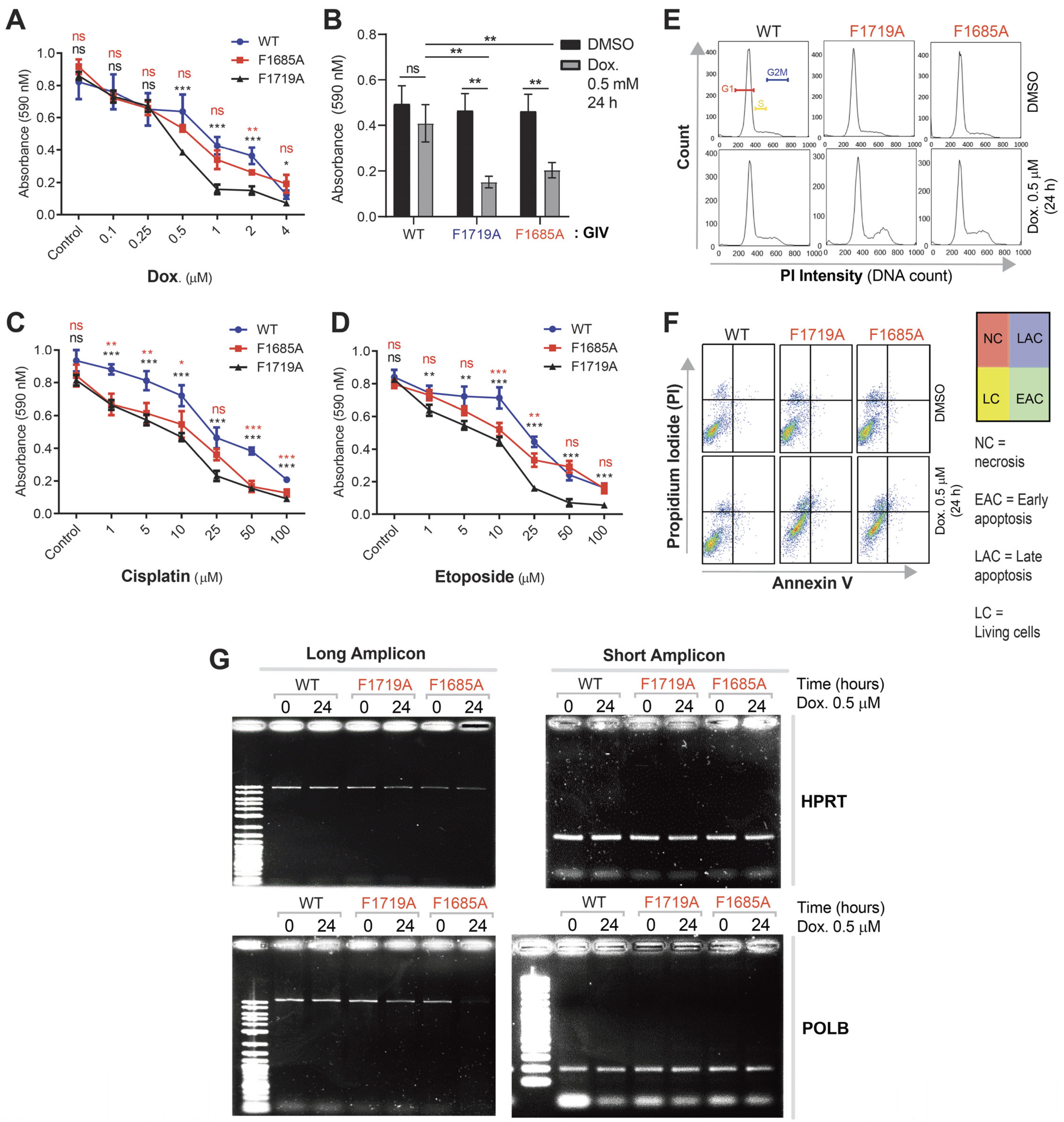

Figure S6 [related to Figure 5]. DNA damage repair response is impaired in cells expressing mutant GIV that cannot bind BRCA1 (F1719A) or bind/activate G proteins (F1685A). A-D. Line (A, C, D) and bar (B) graphs display metabolically active GIV-WT (blue), GIV-F1685A (red) and GIV-F1719A (black) cells that survived various doses of Doxorubicin (A-B), Cisplatin (C) or Etoposide (D), as determined by *MTT* tetrazolium assay (see *Methods*). Data displayed as mean ± S.E.M. and t-test was used to determine significance. (*; p ≤ 0.05; **p < 0.01, ***p < 0.001. ns = not significant). See also **Fig 5C** for the table of IC50 values. **E**. Histograms show the percentage of cells at various stages of cell cycle (G1, S and G2/M) after challenged with Dox or vehicle control (DMSO). See bar graphs in **Fig 5D** for quantification. F. Necrosis (NC), apoptotic (early, EAC; late, LAC; or combined) or living cells (LC) were quantified after challenged with either Dox or vehicle control (DMSO), as assessed by annexin V staining and flow cytometry. Color coded quadrants are labeled. **G**. Long amplicon qPCR (LA-QPCR) was used to evaluate genomic DNA SB levels in control vs. GIV KO cells. Representative full-length gels showing PCR-amplified fragments of the *HPRT* (E, top panel) and *POLB* (E, bottom panel) genes.

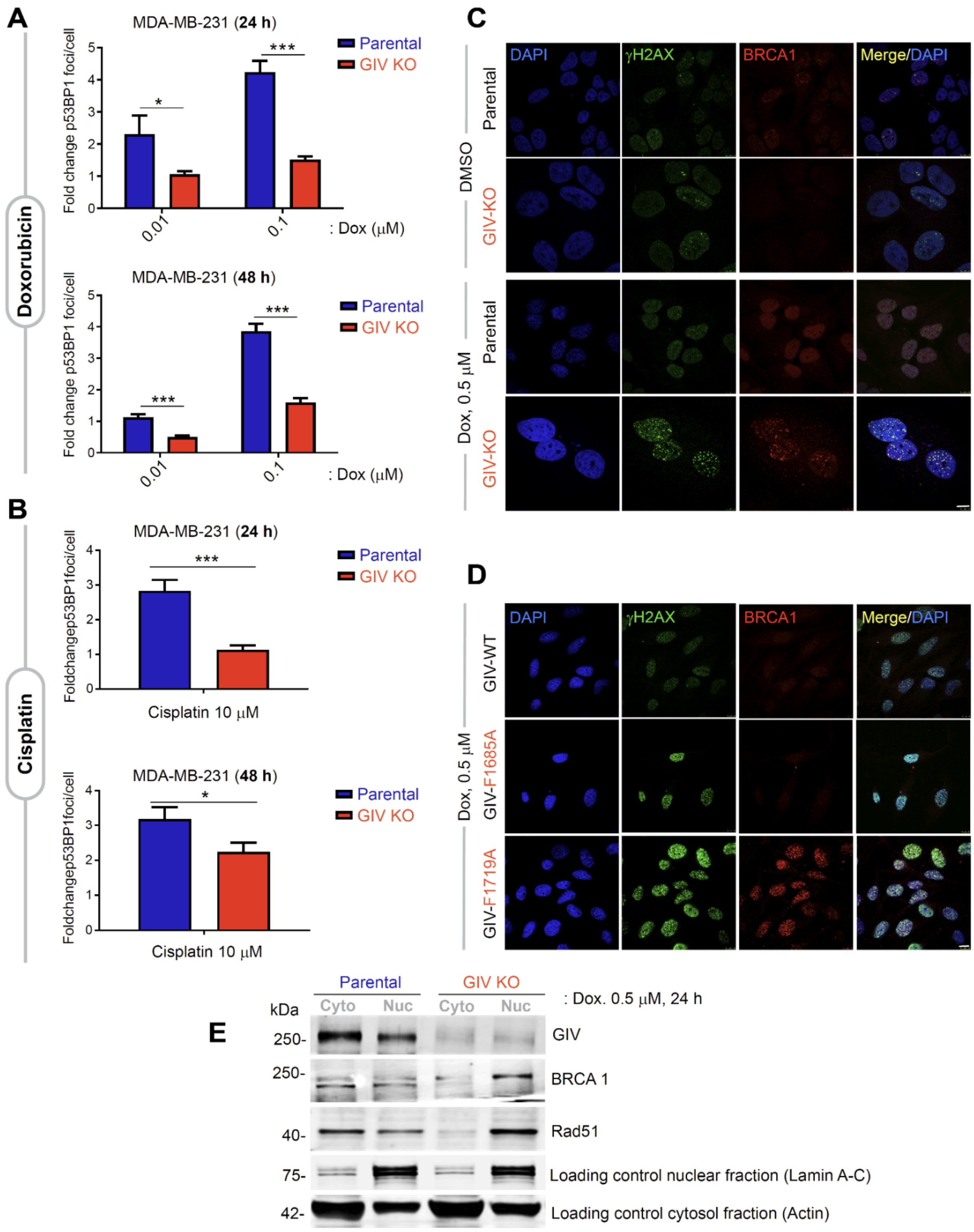

Figure S7 [related to Figure 6]. GIV inhibits the localization of BRCA1 to sites of DNA damage. A-B. Bar graphs display the fold change in the number of bright foci of 53BP1 in parental and GIV KO MDA-MB-231 cells stably expressing mApple-53BP1 reoprter (which detects NHEJ) upon challenge with the indicated concentrations of Doxorubicin (A) or Cisplatin (B). Data displayed as mean ± S.E.M. and t-test to determine significance. (*; $p \leq 0.05$; ***; $p \leq 0.001$). See also **Fig 6F-H** for 53BP1 reporter studies on parental and GIV KO HeLa cells. **C-D**. Control (parental) and GIV-depleted (GIV KO) HeLa cells (**C**) or GIV-depleted HeLa cells stably expressing WT or mutant GIV constructs (D) were challenged with Dox or vehicle control (DMSO) prior to being fixed and co-stained for ɣ2H2AX (green) and BRCA1 (red) and analyzed by confocal microscopy. Representative images are shown (scale bar = 15 μm). **E**. Equal aliquots of nuclear (Nuc) and cytosolic (cyto) fractions prepared from Dox-challenged HeLa parental or GIV KO cells were probed for the indicated proteins by immunoblot. Loading controls are indicated.

SUPPLEMENTARY TABLES

Supplementary Table 1 [related to Figure 1]: Gene ontology (GO) cellular component analysis for GIV interacting proteins, as determined by DAVID GO. Data are available via ProteomeXchange with identifier PXD022601.

Category	Term	Count	%	P Value	Genes
**GOTERM_CC_DIRECT**	GO:0070062~extracellular exosome	69	38.3333333	5.16E-14	HIST2H2AA3, POTEKP, S100A7, SLC7A8, RPS2, CDSN, ACTG1, AZGP1, HIST2H2AB, HEATR5B, HIST1H2BL, HIST2H2AC, SBSN, DMKN, RPL26L1, TUBB6, LTF, H2AFX, TUBB8, TUBB1, AMY2B, TUBA1B, AMY2A, IGLL5, MYO6, ACTA1, HIST1H1B, H2AFJ, FLNB, ALDH16A1, FLNA, LAP3, ADGRV1, PSMA3, RPS13, HSPB1, SERPINB4, DST, AMY1A, ACTBL2, HIST1H2AA, HIST1H2AD, RPS15A, FAM65A, POTEJ, PSMB6, TGM3, SERPINB12, POTEF, POTEE, ENO1, PTPRC, ACACA, RPL26, DDX5, FBL, KRT32, COL5A1, KRT38, RPL23, CASP14, HIST1H2AI, HIST1H2AH, HIST1H2AJ, MUC19, IGLC2, IGLC1, FABP5, DMBT1
**GOTERM_CC_DIRECT**	GO:0000786~nucleosome	14	7.77777778	6.21E-12	HIST2H2AA3, HIST1H2AA, HIST1H1B, HIST1H2AD, HIST1H1A, H2AFJ, HIST2H2AB, HIST1H2BL, HIST2H2AC, HIST1H2AI, HIST1H2AH, H2AFX, HIST1H2AJ, H3F3C
**GOTERM_CC_DIRECT**	GO:0000790~nuclear chromatin	14	7.77777778	4.94E-08	HIST2H2AA3, HIST1H2AA, HIST1H1B, HIST1H2AD, HIST1H1A, H2AFJ, HIST2H2AB, HIST2H2AC, HIST1H2AI, HIST1H2AH, H2AFX, HIST1H2AJ, RUNX2, NCOR1
**GOTERM_CC_DIRECT**	GO:0031012~extracellular matrix	16	8.88888889	1.94E-07	ACTG1, NES, COL7A1, RPL23, CASP14, SBSN, COL3A1, RPS15A, RPS13, HSPB1, COL2A1, COL1A1, DDX5, FLNB, COL5A1, FLNA
**GOTERM_CC_DIRECT**	GO:0022625~cytosolic large ribosomal subunit	7	3.88888889	4.97E-05	RPL17, RPL23, SURF6, RPL26, RPL3L, RPL26L1, RPL36AL
**GOTERM_CC_DIRECT**	GO:0072562~blood microparticle	9	5	1.16E-04	ACTG1, IGLC7, ACTA1, F13A1, IGLC2, POTEF, IGLL5, IGLC1, POTEE
**GOTERM_CC_DIRECT**	GO:0030018~Z disc	8	4.44444444	1.49E-04	RYR3, AHNAK2, HSPB1, FLNC, FLNB, JPH1, DST, FLNA
**GOTERM_CC_DIRECT**	GO:0042571~immunoglobulin complex, circulating	4	2.22222222	7.66E-04	IGLC7, IGLC2, IGLL5, IGLC1
**GOTERM_CC_DIRECT**	GO:0005925~focal adhesion	12	6.66666667	0.0015006	LAP3, ACTG1, PTPRC, RPL23, S100A7, RPS13, HSPB1, FLNC, RPS2, FLNB, DST, FLNA
**GOTERM_CC_DIRECT**	GO:0005581~collagen trimer	6	3.33333333	0.00198247	COL7A1, COL27A1, COL3A1, COL2A1, COL1A1, COL5A1
**GOTERM_CC_DIRECT**	GO:0005615~extracellular space	25	13.8888889	0.00262843	ACTBL2, COL3A1, COL2A1, ACTG1, AZGP1, POTEJ, DMXL2, COL7A1, LTF, LCN1P1, SERPINB12, AMY2A, POTEF, POTEE, ENO1, ACTA1, PLEKHH3, HSPB1, SERPINB4, COL1A1, IGLC2, IGLC1, TNFAIP2, AMY1A, DMBT1
**GOTERM_CC_DIRECT**	GO:0005913~cell-cell adherens junction	10	5.55555556	0.00423766	EIF4G1, MYO6, PSMB6, KIAA1524, AFDN, DLG5, RPS2, FLNB, FLNA, ENO1
**GOTERM_CC_DIRECT**	GO:0015629~actin cytoskeleton	8	4.44444444	0.00534081	BAIAP2L2, ACTA1, CARMIL2, ACACA, WIPF1, FLNB, DST, FLNA
**GOTERM_CC_DIRECT**	GO:0005604~basement membrane	5	2.77777778	0.00719829	COL7A1, COL2A1, DST, COL5A1, USH2A
**GOTERM_CC_DIRECT**	GO:0015934~large ribosomal subunit	3	1.66666667	0.00781963	RPL17, RPL26, RPL26L1
**GOTERM_CC_DIRECT**	GO:0005737~cytoplasm	66	36.6666667	0.01040164	SCN3A, S100A7, EHHADH, SLC7A8, HLCS, RPS2, ANKRD17, HIST1H2BL, ANKRD12, TUBB6, LTF, AFDN, DLG5, TUBB8, TPR, TUBB1, USH2A,

					MYO6, HERC2, FLNC, FLNB, CDKL5, FLNA, LAP3, EIF4G1, NCOA2, CARMIL2, KIAA1524, TBCD, ADGRV1, PSMA3, HSPB1, SERPINB4, DST, ACTBL2, KMT2A, SSH1, RPS15A, DISP3, DNAH7, NCKAP5L, FAM65A, PSMB6, PLCH2, HNRNPF, CHD1, TGM3, AHNAK2, SERPINB12, RUNX2, ENO1, RGPD5, NES, MKI67, EPB41, ACACA, BRCA1, HNRNPH2, TEX15, RPL23, CASP14, TUBAL3, HNRNPH1, TOB2, FABP5, DMBT1
**GOTERM_CC_DIRECT**	GO:0005874~microtubule	9	5	0.0109175	EHHADH, TBCD, SYNJ1, TUBAL3, TUBB6, TUBB8, DNAH7, TUBB1, TUBA1B
**GOTERM_CC_DIRECT**	GO:0016020~membrane	33	18.3333333	0.01148686	WRNIP1, SLC7A8, RPS15A, RPS2, ACTG1, ANKRD17, HEATR5B, HNRNPF, GYS1, RANBP2, NBEAL2, ENO1, PTPRC, MYO6, MKI67, RPL26, HERC2, DDX5, ALDH16A1, FBL, FLNA, EIF4G1, HNRNPH2, RPL23, CARMIL2, ADGRV1, SNRNP200, RPL3L, RPS13, HNRNPH1, NCOR1, DMBT1, SUCO
**GOTERM_CC_DIRECT**	GO:0002169~3-methylcrotonyl-CoA carboxylase complex, mitochondrial	2	1.11111111	0.01922244	MCCC2, MCCC1
**GOTERM_CC_DIRECT**	GO:1905202~methylcrotonoyl-CoA carboxylase complex	2	1.11111111	0.01922244	MCCC2, MCCC1
**GOTERM_CC_DIRECT**	GO:0005634~nucleus	66	36.6666667	0.02310717	HIST2H2AA3, RPL17, S100A7, RPS2, ACTG1, ANKRD17, HIST2H2AB, AZGP1, HIST1H2BL, HIST2H2AC, TUBB6, LTF, H2AFX, TPR, RPL36AL, MYO6, H2AFJ, HERC2, FLNA, CDKL5, LAP3, EIF4G1, NCOA2, PSMA3, SNRNP200, HSPB1, RPS13, NSD1, DST, HIST1H2AA, KMT2A, HIST1H2AD, WRNIP1, KMT2C, TNFRSF1B, PSMB6, HNRNPF, CEBPZ, CHD1, AHNAK2, FAM71A, RUNX2, ENO1, ASXL3, MKI67, EPB41, ACACB, DDX5, TET2, VSX1, POLR3E, BRCA1, FBL, DLX1, TEX15, HNRNPH2, CASP14, FLG, PPRC1, HIST1H2AI, HIST1H2AH, HIST1H2AJ, ZNF385C, HNRNPH1, NCOR1, TOB2
**GOTERM_CC_DIRECT**	GO:0005882~intermediate filament	5	2.77777778	0.02400783	KRT38, NES, FLG, DST, KRT32
**GOTERM_CC_DIRECT**	GO:0005856~cytoskeleton	9	5	0.02794319	ACTBL2, ACTG1, POTEKP, SSH1, EPB41, PLEKHH3, HSPB1, FLNC, DST
**GOTERM_CC_DIRECT**	GO:0005938~cell cortex	5	2.77777778	0.03146272	MYO6, FLNB, POTEF, DST, FRY
**GOTERM_CC_DIRECT**	GO:0031941~filamentous actin	3	1.66666667	0.0359069	ACTG1, MYO6, FLNA
**GOTERM_CC_DIRECT**	GO:0001652~granular component	2	1.11111111	0.03807745	SURF6, FBL
**GOTERM_CC_DIRECT**	GO:0005788~endoplasmic reticulum lumen	6	3.33333333	0.03878772	COL7A1, COL27A1, COL3A1, COL2A1, COL1A1, COL5A1
**GOTERM_CC_DIRECT**	GO:0030529~intracellular ribonucleoprotein complex	5	2.77777778	0.04297894	HNRNPH2, HNRNPF, SNRNP200, DDX5, BRCA1
**GOTERM_CC_DIRECT**	GO:0019898~extrinsic component of membrane	4	2.22222222	0.04504762	EPB41, TPR, NBEAL2, DMBT1

Supplemental Information 2 [related to Figure 1]: Gene ontology (GO) molecular function analysis for GIV interacting proteins, as determined by DAVID GO. Data are available via ProteomeXchange with identifier PXD02260.

Category	Term	Count	%	PValue	Genes
GOTERM_MF_DIRECT	GO:0009374~biotin binding	4	2.22222222	7.35E-06	MCCC1, HLCS, ACACB, PCCA
GOTERM_MF_DIRECT	GO:0004075~biotin carboxylase activity	4	2.22222222	7.35E-06	MCCC1, ACACA, ACACB, PCCA
GOTERM_MF_DIRECT	GO:0005200~structural constituent of cytoskeleton	9	5	7.46E-06	ACTG1, ACTA1, EPB41, EHHADH, TUBAL3, TUBB6, TUBB8, TUBB1, TUBA1B
GOTERM_MF_DIRECT	GO:0044822~poly(A) RNA binding	25	13.8888889	8.79E-05	RPL17, SURF6, KMT2C, RPS15A, RPS2, ANKRD17, HNRNPF, CEBPZ, TPR, ENO1, MKI67, HIST1H1B, RPL26, DDX5, FLNB, FLNA, FBL, EIF4G1, HNRNPH2, RPL23, SNRNP200, PPRC1, RPS13, HSPB1, HNRNPH1
GOTERM_MF_DIRECT	GO:0048407~platelet-derived growth factor binding	4	2.22222222	1.16E-04	COL3A1, COL2A1, COL1A1, COL5A1
GOTERM_MF_DIRECT	GO:0003723~RNA binding	16	8.88888889	1.47E-04	SURF6, SYNJ1, RPL26, RPS15A, RPS2, FBL, BRCA1, EIF4G1, ANKRD17, HNRNPH2, HNRNPF, RPL3L, FBLL1, RANBP2, ZNF385C, HNRNPH1
GOTERM_MF_DIRECT	GO:0003989~acetyl-CoA carboxylase activity	3	1.66666667	2.47E-04	ACACA, ACACB, PCCB
GOTERM_MF_DIRECT	GO:0004556~alpha-amylase activity	3	1.66666667	8.12E-04	AMY2B, AMY2A, AMY1A
GOTERM_MF_DIRECT	GO:0003735~structural constituent of ribosome	9	5	9.95E-04	RPL17, RPL23, RPL26, RPL3L, RPL26L1, RPS13, RPS15A, RPS2, RPL36AL
GOTERM_MF_DIRECT	GO:0003677~DNA binding	29	16.1111111	0.00114984	HIST2H2AA3, HIST1H2AA, SSH1, KMT2A, SURF6, WRNIP1, KMT2C, HIST1H2AD, HIST2H2AB, HIST1H2BL, HIST2H2AC, CEBPZ, LTF, CHD1, H2AFX, RUNX2, ENO1, ASXL3, MKI67, H2AFJ, ATR, TET2, VSX1, BRCA1, DLX1, HIST1H2AI, HIST1H2AH, HIST1H2AJ, NCOR1
GOTERM_MF_DIRECT	GO:0034987~immunoglobulin receptor binding	4	2.22222222	0.00165945	IGLC7, IGLC2, IGLL5, IGLC1
GOTERM_MF_DIRECT	GO:0005201~extracellular matrix structural constituent	5	2.77777778	0.00326918	COL27A1, COL3A1, COL2A1, COL1A1, COL5A1
GOTERM_MF_DIRECT	GO:0019899~enzyme binding	10	5.55555556	0.00352893	EHHADH, HIST1H2AI, H2AFX, HLCS, SERPINB4, SERPINB12, DDX5, RPS2, BRCA1, PCCA
GOTERM_MF_DIRECT	GO:0098641~cadherin binding involved in cell-cell adhesion	9	5	0.00518765	EIF4G1, MYO6, PSMB6, KIAA1524, AFDN, RPS2, FLNB, FLNA, ENO1
GOTERM_MF_DIRECT	GO:0005524~ATP binding	24	13.3333333	0.00899823	TRPM4, ACTBL2, POTEKP, MYO6, MYH15, ACTA1, MKI67, WRNIP1, ACACA, HLCS, ACACB, **ATR,** DDX5, DNAH7, CDKL5, ACTG1, MCCC2, MCCC1, SNRNP200, CHD1, PCCB, RUNX2, PCCA, CACNA1B
GOTERM_MF_DIRECT	GO:0046982~protein heterodimerization activity	11	6.11111111	0.01035566	HIST2H2AA3, HIST2H2AB, HIST1H2AA, HIST1H2BL, HIST1H2AD, HIST2H2AC, HIST1H2AI, HIST1H2AH, H2AFX, H3F3C, H2AFJ
GOTERM_MF_DIRECT	GO:0003779~actin binding	8	4.44444444	0.01394253	MYH15, MYO6, SSH1, EPB41, POF1B, WIPF1, FLNB, DST
GOTERM_MF_DIRECT	GO:0003823~antigen binding	5	2.77777778	0.01466781	AZGP1, IGLC7, IGLC2, IGLL5, IGLC1
GOTERM_MF_DIRECT	GO:0004658~propionyl-CoA carboxylase activity	2	1.11111111	0.01816268	PCCB, PCCA
GOTERM_MF_DIRECT	GO:0004485~methylcrotonoyl-CoA carboxylase activity	2	1.11111111	0.01816268	MCCC2, MCCC1

GOTERM_MF_DIRECT	GO:1990259~histone-glutamine methyltransferase activity	2	1.11111111	0.01816268	FBLL1, FBL
GOTERM_MF_DIRECT	GO:0008649~rRNA methyltransferase activity	2	1.11111111	0.02712073	FBLL1, FBL
GOTERM_MF_DIRECT	GO:0003824~catalytic activity	6	3.33333333	0.02908	EHHADH, TGM3, AMY2B, NRDC, AMY2A, AMY1A
GOTERM_MF_DIRECT	GO:0005516~calmodulin binding	6	3.33333333	0.02966422	TRPM4, MYH15, MYO6, EPB41, RYR3, DDX5
GOTERM_MF_DIRECT	GO:0018024~histone-lysine N-methyltransferase activity	3	1.66666667	0.04917698	KMT2A, KMT2C, NSD1

Supplementary Table 3 [related to Figures 2 and **5]:** Summary of phenotypes observed in various cell lines used in this study.

	Property	Survival	Cell Cycle Phase arrest	Necrosis	Apoptosis	HR	NHEJ	Mutated DNA
GIV +/+	GIV can scaffold G protein and BRCA1	+	S	-	-	-	+	-
GIV -/-	G protein and BRCA1 disconnected	-	G2/M	+	+	+	-	+
GIV- WT	GIV can scaffold G protein and BRCA1	+	S	-	-	-	+	-
GIV-F1719	GIV binds and activates G proteins but does not bind BRCA1.	-	G2/M	+	+	+	-	+
GIV-F1685	GIV binds BRCA1 but cannot bind/activate G proteins.	-	S and G2M	+	+	+	-	+

Supplementary Table 4 [related to Figure 6]: Summary of Predicted Nuclear Import and Export Sequences in GIV/Girdin

Elm Name	Instances (Matched sequence)	Positions	Elm Description	Cell Compartment	Consensus Pattern	P value
TRG_NES_CRM1_1	QLKAKLHDMEMERD<br>ELLQKKITNLKITCE<br>QQLESELQDLEME<br>QLESELQDLEME<br>QLEDLEKMLKVEQE<br>ENLLDEVMKSLSVSSD	386-399 [A]<br>642-656 [A]<br>780-792 [A]<br>781-792 [A]<br>1211-1224 [A]<br>1704-1719 [A]	Some proteins re-exported from the nucleus contain a Leucine-rich nuclear export signal (Shameer et al.) binding to the CRM1 exportin protein.	nucleus, cytosol	([DEQ].{0,1}[LIM].{2,3}[LIVMF][^P]{2,3}[LMVF].[LMIV].{0,3}[DE])|([DE].{0,1}[LIM].{2,3}[LIVMF][^P]{2,3}[LMVF].[LMIV].{0,3}[DEQ])	7.626e-04
TRG_NLS_MonoExtC_3	NRKLKK	672-677 [A]	Monopartite variant of the classical basically charged NLS. C-extended version.	nucleus, Nuclear pore, NLS-dependent protein nuclear import complex	[^DE]((K[RK])|(RK))(([^DE][KR])|([KR][^DE]))(([PKR])|([^DE][DE]))	7.252e-04
TRG_NLS_MonoExtN_4	RENRKLKK<br>KENKRLRQ	670-677 [A]<br>836-843 [A]	Monopartite variant of the classical basically charged NLS. N-extended version.	nucleus, Nuclear pore, NLS-dependent protein nuclear import complex	(([PKR].{0,1}[^DE])|([PKR]))((K[RK])|(RK))(([^DE][KR])|([KR][^DE]))[^DE]	1.276e-03
